# Melt Spinning of Thermoplastic Polyurethane-Based Bulk Ionofibers Filled with Carbon Nanotubes

**DOI:** 10.1021/acsapm.5c00286

**Published:** 2025-05-23

**Authors:** Claude Huniade, Aurélie Cayla, Tariq Bashir, Nils-Krister Persson

**Affiliations:** 1 Polymeric E-textiles, The Swedish School of Textiles, University of Borås, Borås 501 90, Sweden; 2 University of Lille, ENSAIT, ULR 2461 - GEMTEX - Génie et Matériaux Textiles, Lille F-59000, France

**Keywords:** melt-spinning, conductive polymer composites, ionic liquids, carbon nanotubes, monofilaments, i-textiles

## Abstract

Ionotronic textiles or i-textiles offer in-air electrochemical applications and sensing due to their ionic character, mimicking phenomena of organisms. To manufacture different i-textiles with unique functions and characteristics, it is necessary to have a range of ionically conductive textile fibers or ionofibers to choose from. However, their means of production are not sufficiently explored to provide knowledge that meets the fabric manufacturing needs. For a textile application, surface functionalization is usually explored as a convenient way to build upon an already known textile material. In contrast, bulk functionalization allows for superior production rate, versatility, and durability. Additionally, the use of the synergy between ionic liquids and carbon nanotubes is seldom explored. Therefore, in this study, melt spinning is investigated regarding the use of an ionic liquid (IL) initially without and ultimately with multiwalled carbon nanotubes (CNTs) for the tailoring of the electrical and mechanical properties of ionofibers. Based on thermoplastic polyurethane (TPU) elastomers, IL-containing pellets are prepared using 1-ethyl-3-methylimidazolium trifluoromethanesulfonate (EMIm OTf) at different weight ratios. About the melt-spun monofilaments, their extrusion temperatures, their morphology through scanning electron microscopy with energy-dispersive X-ray, their fiber conductivity through electrochemical impedance spectroscopy and cyclic voltammetry, and their tensile properties are investigated. An optimum of the ratios of IL and CNTs is observed for the melt-spinning process, which results in fiber conductivities within the range of 10^–2^ μS cm dtex^–1^. Compared to a monofilament melt-spun with no IL and a CNT weight ratio above percolation threshold, the fiber conductivity is twice higher due to its intricate segregated network. Thus, this industrial textile-compatible process offers an alternative within the development of ionotronic fabrics.

## Graphical abstract

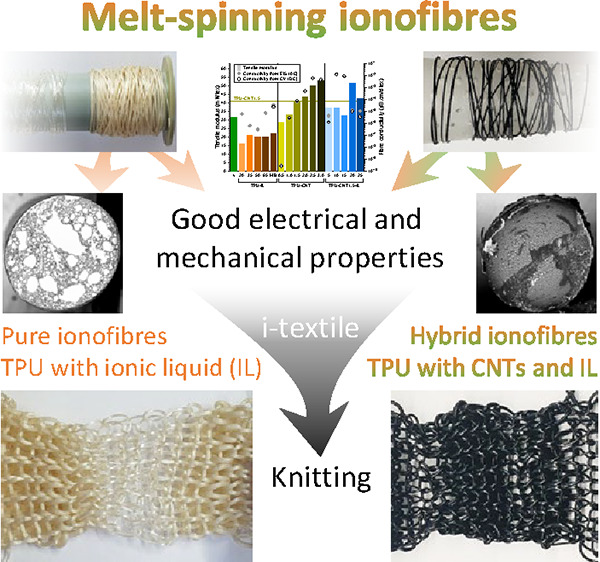

## 1 Introduction

Textiles have a long history of thousands of years with extensive knowledge and technology on producing soft, conformable, and compliant materials. Lately, ionofibers,^1^ i.e., ionically conductive textile fibers, offer promising perspectives for ionotronic textiles or i-textiles with enhanced textile properties and in-air electrochemical applications. Their applications as ionotronics include conformal energy storage devices, bioelectronic interfaces, photovoltaic devices, electroluminescent devices, textile sensors, and textile muscles. As an example, textile muscles based on electroactive polymers (EAPs) require the EAPs and an ion-source/-sink in fiber form to manufacture addressable fabrics out of them.^2^ The fiber form can be achieved in two ways: by functionalizing the surface of pre-existing textile fibers or by forming bulk filaments with an appropriate process.

However, the production rate of ionofibers, which needs to be high when being a raw material for manufacturing fabrics, is still limited. The production rate when coating has the solidification/curing mechanism of the precursor as a bottleneck, whereas polymer spinning relies directly on fiber-forming properties of the compound. Thus, from a scientific and industrial standpoint, there is interest in exploring the fiber-forming ability of bulk ionofibers to better understand the benefits and drawbacks of each approach.

The melt-spinning process is the most commonly used fiber-forming process for producing synthetic filaments with thermoplastic polymers.^3^ 1-Ethyl-3-methyl imidazolium bis­(trifluoromethylsulfonyl)­imide (EMIm TFSI), an imidazolium-based ionic liquid (IL), was shown to be compatible with thermoplastic polyurethane (TPU) to produce either surface ionofibers by dip-coating,^4^ or bulk ionofibers by piston-spinning.^5^ However, the melt-spinning of TPU-based bulk ionofibers has not been thoroughly reported and investigated, which is the main focus of this study. Compared to the solvent techniques, melt spinning is more practical and economical on an industrial scale.^3^ Even with nanofillers, this process allows for high production rates without the use of solvents.

Regarding nanofillers, multiwalled carbon nanotubes (CNTs) have already been used to produce conductive polymer composite (CPC) filaments through melt-spinning.^6–15^ Their high aspect ratio typically led to the highest conductivities among carbon fillers.^16^ However, limited supplies, as well as toxicity and processing issues related to the use of CNTs, impair their commercialization and acceptability.^3^ At low weight ratios, CNTs heavily impact the viscosity of polymers. Graphene, i.e., a similar carbon filler, has shown synergy with ILs bringing higher conductivities with lower weight ratios versus the conductive fillers incorporated on their own.^17^ This has been associated with the increased dispersion of conductive carbon fillers due to the strong physical interaction of their π electrons with the imidazolium ions.^18–20^ Such interactions construct efficient pathways for electrical currents that can reduce the inevitable electrochemical losses coming from ohmic drops and overpotentials (which may result in degrading performance over time) when using pure ionofibers in i-textiles.

Previously, results of functionalizing an IL-based coating (an ionogel) on pre-existing core textile filaments, also known as surface ionofibers, were reported.^1^ Here, the aim of the study is to present an alternative to IL-based coatings with the melt-spun bulk ionofibers based on TPU. The synergy between ILs and CNTs as conductive materials in the melt-spun filaments was also explored. Therefore, three kinds of TPU-based monofilaments were produced:

1. first, pure bulk ionofibers with an IL as an ionically conductive medium,
2. for reference, CPC monofilaments with CNTs as an electronically conductive filler,
3. and finally, hybrid bulk ionofibers using both the IL and CNTs.

Their processing in melt-spinning was observed with varied IL weight ratios. The monofilaments were evaluated in terms of morphology as well as mechanical (tensile) and conductive properties. Finally, their processability was shown with a manual knitting machine. This study provides a first attempt with melt-spinning regarding the production of melt-spun ionofibers and explores the synergy between ILs and CNTs as conductive materials.

## 2 Experimental Section

### 2.1 Preparation of the TPU-IL Pellets

The materials used for the preparation of the blends of TPU and IL (TPU-IL) are Elastollan 1185A TPU pellets, dimethyl sulfoxide (DMSO), and 1-ethyl-3-methylimidazolium trifluoromethanesulfonate (EMIm OTf), which were supplied by BASF, Merck, and IoLiTec GmbH (Germany), respectively. The TPU pellets were dried at 40 °C in an Ecocell 111 oven from MMM Medcenter Einrichtungen GmbH (Germany) before use. The other chemicals were used as received.

#### 2.1.1 Varied IL Ratio Batch

Our preparation was inspired from works by Kim et al.,^4,21^ as well as Shi et al.,^5^ with a couple of chemical substitutions. *N*,*N*-Dimethylformamide (DMF) was substituted with DMSO and EMIM TFSI by EMIm OTf because DMSO and EMIm OTf are considered less toxic and thus more sustainable than their counterpart. This is an important consideration for the potential upscaling of the production and end use of the products. TPU pellets were dissolved to produce 25 wt % solutions in DMSO under stirring in a water bath at 70 °C on a magnetic stirrer hot plate for 2–3 h. EMIm OTf was then added to meet the weight ratios of 20, 35, 50, and 65 wt % in TPU for further stirring (70 °C and 1 h). The TPU-IL solutions were then casted on Petri dishes and put to dry in the oven for at least 12 h. After drying, any remaining excess liquid was discarded. The TPU-IL films were then cut into small pellets using scissors. The TPU-IL pellets were further dried apart from each other to avoid clogging in a GPC 1200 chamber furnace from Carbolite Gero (UK/Germany) at 80 °C and finally stored in polypropylene containers right before use. Choline acetate (supplied by IoLiTec GmbH) had also been tried in parallel with EMIm OTf but seemingly degraded with temperature when combined with DMSO. The result of that preparation was a green slurry that did not solidify. This was not further investigated as its use was deemed potentially toxic and of high risk.^22^

#### 2.1.2 TPU-IL Masterbatch

The same procedure was followed for the TPU-IL masterbatch, with the distinction that, for better homogeneity, TPU was dissolved to produce 21 wt % solutions in DMSO instead of 25 wt %. Also, only 65 wt % TPU-IL pellets were prepared in eight parallel processes. These pellets were used for producing the TPU-CNT-IL monofilaments and a TPU-IL reference monofilament.

### 2.2 Preparation of the TPU-CNT Masterbatch

The materials used for the preparation of the TPU-CNT masterbatch were also Elastollan 1185A TPU pellets and NC7000 multiwalled CNTs supplied by Nanocyl (Belgium). The CNTs were incorporated into the TPU at 5 wt % by feeding a Thermo Scientific (USA) Process 11 twin-screw extruder via a glovebox after first feeding it virgin TPU. The screw rotational speed was fixed at 100 rpm. The temperatures set for the die and the different zones of the extruder are available in Table S1 in the Supporting Information. The extrudate was directly fed to a Haake PP1 Pelletizer POSTEX from Thermo Scientific (USA) positioned 2–3 m away from the die.

### 2.3 Thermal Analysis

Thermogravimetric analysis (TGA) and differential scanning calorimetry (DSC) were performed on the pellets using a TA Q2000 and a Q500 instrument from TA Instruments (USA), respectively. For the TGA, the ramp was set to 10.00 °C min^–1^ up to 700.00 °C. For the DSC, a full cycle was of heating and cooling was performed between −20.00 and 240.00 °C with a ramp of 10.00 °C min^–1^.

### 2.4 Melt Spinning of Monofilaments

The different weight ratios of CNT and IL used for the melt spinning on a MiniLab twin-screw extruder from Thermo Haake (Germany) are given in the form TPU-CNT*x*-IL*y* with *x* and *y* in wt %. Table S2 in the Supporting Information illustrates all the different samples prepared. The rotational speed of the screw was fixed at 50 rpm. Due to the low amount of material for each ratio, the material had to be manually fed; thus, the input rate was loosely controlled. The spinneret consisted of a round hole with a diameter of 800 μm. Nonetheless, a motorized belt conveyor was used for drawing the extrudate with a speed of 1.5 m min^–1^, unless stated otherwise. The monofilaments were collected on a spool at the end of the conveyor belt (∼1.5 m). The monofilaments with only varying IL wt % (no CNT) were produced by using suitable pellets from the varied IL ratio batch. For the other monofilaments, polypropylene containers were prepared from the applicable masterbatches and leaving them to dry at 40 °C overnight in a KB240 oven from Binder (Germany) before use.

### 2.5 Storage of the Samples

Since most of the characterization of the monofilaments was done a year after their production, it was necessary to keep track of the storage conditions in case of aging effect. The monofilaments were stored a year in a box in the ambient condition of the laboratory (with seasonal fluctuations observed between 19 and 24 °C and 10–71%RH) before characterization, unless stated otherwise.

### 2.6 Linear Density

In the textile industry, the linear density or fineness gives consistent information from the molecular to the macroscopic level.^23^ The fiber fineness is conveniently expressed in decitex (dtex). A tex is defined as 1 g km^–1^ or 10^–6^ kg m^–1^ in SI units, so a dtex equals to 10^–7^ kg m^–1^.^1^ The linear density was calculated from the weight of a single sample to the precision of 0.1 mg (1 dtex for 100.0 cm). Due to the loosely controlled input rate, a representative sample was selected with a length as long as 100.0 cm. The samples were measured in the same laboratory previously mentioned at ambient temperature and relative humidity (20 °C and 27%RH).

### 2.7 SEM with EDX Mapping

The scanning electron microscopy (SEM) images were obtained using an FEI (USA) Quanta 200 FEG ESEM equipped with an Oxford X-max 80 energy-dispersive X-ray (EDX) detector in high vacuum mode. The cryofractured samples were prepared by dipping them in liquid nitrogen before hitting them with a scalpel for a clean cut. They were then attached on SEM stubs with double-sided carbon tapes. This was performed for cross sections and longitudinal sections. The results of the EDX map sums of fluor and sulfur were processed into pseudocolored images with a look-up table as the following: black for pixel value 0, red for 1, yellow to green gradient from 2 to 7.

### 2.8 Tensile Testing

Behavior and ultimate tensile properties of the samples were studied with a Mesdan (Italy) 2512A tensile tester in a conditioned room (21 °C and 65%RH). The tensile tests were performed using pneumatic yarn grips equipped with a load cell of 0.1 kN (resolution: 1 cN), at the speed of 100% elongation min^–1^. The samples were clamped with a pretension lower than 10 cN, which was enough to straighten the monofilament. The gauge length was 50 mm. The data recording was performed every 0.1 mm elongation. The initial slopes were extracted through a linear fitting of the force–strain curves between 0 and 2% strain.

### 2.9 Fiber Conductivity

Electrical conductivity tests were conducted on an Autolab PGSTAT204 potentiostat/galvanostat instrument with a FRA32M module from Metrohm (Switzerland). The samples were set on a two-point probe at ambient temperature and relative humidity (20 °C and 27%RH unless stated otherwise). The probe consisted of cylinder electrodes held parallel and horizontal with a 20 mm space between their axes. The fibers were held down by their end with an attaching clip and weight (103.6 g) each on each end. The excitation signal for the electrochemical impedance spectroscopy (EIS) was of sine wave type of an amplitude of 0.5 V_PK_ around 0 V in the frequency range of 1 MHz to 0.1 Hz for most samples with a step of 15 points per decade, an integration time of 1.5 s, and three integration cycles. Due to too many scattered signals for the varied IL ratio batch and TPU-CNT0.5, the integration time for their EIS measurement was 15 s instead. The excitation signal for the cyclic voltammetry (CV) was of staircase type between −5 and +5 V with a start potential of 0 V, a scan rate of 0.01 V s^–1^, and a step of 0.005 V. The scanning was done three times. The electrical properties were evaluated by calculating the fiber conductivity σ_f_ expressed in μS cm dtex^–1^ (analogous to S cm^–1^) using eq 1, with the length between the electrodes *L*, the resistance *R*, and the fineness of the sample λ_m_.^1^

$$\sigma_{f}=\frac{L}{R\times \lambda_{m}}$$ 1

For the EIS, the resistances were extracted from the diameter of the semicircles (i.e., the charge-transfer resistance *R*_ct_ of a Randles circuit) on the resulting Nyquist plot. For the cyclic voltammetries, the resistances were extracted from the average of the slope going from 1 to −1 V of each scan. The allometric fittings were done on part of the data with the potential starting at 0 V and ending at 5 V.

### 2.10 Knitting

Regarding the processability on a manual knitting machine, an E4 double bed flat knitting machine type JBO from Stoll (Germany) was used. The latch needles were set for a knitting width of 40 mm, and the stitch cams at 12 mm on both needle beds. The elasticity and friction of the materials required the monofilaments to be fed and tensioned manually (without the usual tension mast).

### 2.11 Statistical Analysis

Microsoft Excel was used for statistical analysis. The results are either expressed as single measured/calculated values, or mean values with 95% confidence interval based on the standard deviation of the mean. Due to small sample sizes (*n* < 30), this resulted in limited statistical power to assess significant differences.^24^ Therefore, confidence intervals were provided for all of the mean values calculated directly from measurements.

## 3 Results and Discussion

### 3.1 Extrusion Temperatures

The extrusion temperatures for the melt-spun ionofibers were at first sight highly dependent on the quality of the preparation of the TPU-IL pellets. The current preparation procedure was deemed to be not reliable enough to ensure that the exact weight ratio used was kept in the pellet format. On top of this, the heating and drying parts of the procedure may have resulted in damaged pellets of two weight ratios. This caused unexpected behavior when melt spinning as seen with the required extrusion temperatures dropping from 170 to 140 or 157 °C (Table S3 in the Supporting Information) despite the thermal analysis of single pellets not showing this discrepancy (Figures 1 and 2). For both TPU-IL35 and TPU-IL50, the extrudates connected to themselves on the collecting spool before fully solidifying. In the most extreme case (TPU-IL50), the crystallization finished on the spool with TPU chains that crystallized from one surface to another. Separating the merged monofilaments required us to stretch them a little, which could have impacted their characterization. When melt spinning from TPU-IL pellets only, the most noticeable effects were the need for lower extrusion temperatures and the slower crystallization. The former is typically around the same extrusion temperature of virgin TPU (vTPU); the latter was also observed with the shift of the crystallization peaks on the DSC curves (Figure 2). Regarding the melting process, TPU shows multiple zones of heat absorption due to hard and soft segments (see the enlarged view of Figures S1a and S2, Supporting Information). Therefore, the endothermic temperatures are reported in Table 1. However, the endothermic or crystallization temperatures did not display any clear dependency on the concentration of IL. They may be more dependent on the preparation procedure of the TPU-IL pellets since the thermal analysis of different TPU-IL65­(MB) pellets was slightly more distinct than pellets from the varied IL ratio batch (see Figure S3, Supporting Information). TPU-IL65­(MB) had overall a lower crystallization rate than the varied IL ratio batch and even lower than vTPU.

**Figure 1:**
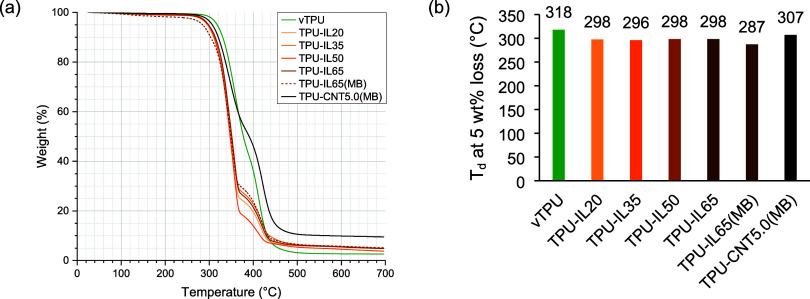
(a) TGA curves under a nitrogen purge (60 mL min^–1^). (b) Degradation temperature at 5 wt % loss. The confidence interval at 95% for TPU-IL65­(MB) was 2.04 °C.

**Figure 2:**
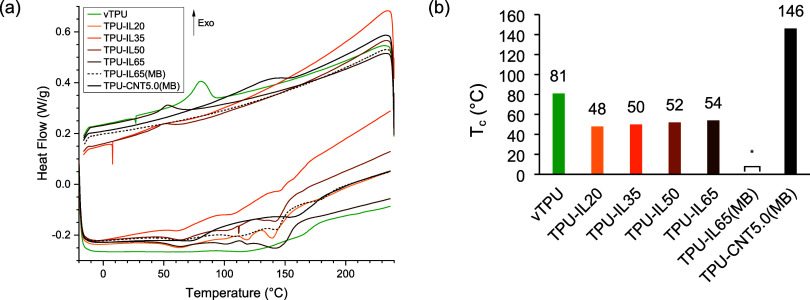
(a) DSC curves of the prepared pellets. Additional plots with the enlarged view of the endothermic peaks, the crystallization peaks, and with the full range of TPU-IL65­(MB) are available in the Supporting Information. (b) Extracted crystallization temperatures from peaks. *: No noticeable peak.

**Table 1:** Endothermic Temperatures Extracted from the Peaks of the DSC Curves during Heating

	T1 (°C)	T2 (°C)	T3 (°C)	T4 (°C)	T5 (°C)
vTPU	74	100	115	138	165
TPU-IL20	63	100	119	139	
TPU-IL35	60	106	146	157	166
TPU-IL50	63	104	133	142	165
TPU-IL65	54	101	119	144	
TPU-IL65(MB)^a^	63	109	121	143	178
TPU-CNT5.0(MB)	71			159	

a Extracted from one of the pellets tested.

When melt-spinning TPU-IL and TPU-CNT pellets together, the extrusion temperature was required to be high like vTPU or TPU-CNT samples for good fluidity as CNT and IL mixtures can become viscous.^18^ However, when the concentration of IL was more important (TPU-CNT1.5-IL20 and higher), the extrusion temperature had to be lowered since the extrudates became too fluid and not solidifying fast enough. This led to instabilities of the extrudate, i.e., knots. Thus, nondrawn extrudates were produced starting with TPU-CNT1.5-IL20 up to TPU-CNT1.5-IL40 (Table S3). From TPU-CNT1.5-IL30, the more pronounced heterogeneity of the spinning melt or the presence of knots made the drawing of the extrudates impracticable.

Due to the loosely controlled input rate, the linear density and quality of the extrudates were visibly variating. Therefore, instead of the usual random sampling and for more accurate representation of the whole extrudate, a single sample with as little visual variation and as long as possible (up to 100.0 cm) was selected per extrudate for their characterization.

### 3.2 Morphology

SEM images were used together with EDX mapping to observe the morphology and the localization of the IL within ionofibers. Backscattered electrons were ideal to identify IL-heavy phases (in white), as confirmed by the concentration of fluor and sulfur from their EDX sum map (Figure 3 and Figure S4, Supporting Information). These marble-like IL-heavy phases were presumed entrapped IL since they could be partly extracted by capillarity in contact with the carbon tapes the samples are attached onto. The marble-like IL-heavy phases of TPU-IL50 looked collapsed due to the stretch when separating the merged monofilaments. The longitudinal section of TPU-IL65­(MB) (Figure 4) also showed these marble-like phases like the cross section but to a lesser extent partly due to the slower crystallization. For some hybrid ionofibers, the opposite was clearly observable as there were more, though smaller, IL-heavy phases due to CNTs accelerating nucleation and crystal growth (Figure S5 in the Supporting Information).^25^ Also, the concentration of IL impacted the quality of the marbling in the hybrid ionofibers as better seen on their cross sections (Figure S6 in the Supporting Information). Especially for TPU-CNT1.5-IL10 and TPU-CNT1.5-IL15, the dark phases at the edges of the IL-heavy phases, which are presumably CNT-containing paths, formed a more segregated network (Figure 5 and Figure S7 in the Supporting Information).^26^ Its formation can be associated with the strong physical interaction of the π electrons of the CNTs with the imidazolium ions.^18–20^ Comparing TPU-IL20 with TPU-CNT1.5-IL20 (Figure 3 and Figure S6e in the Supporting Information), the inclusion of CNTs caused the IL-heavy phases to be smaller, thus indicating an increased compatibility between TPU and the IL.

**Figure 3:**
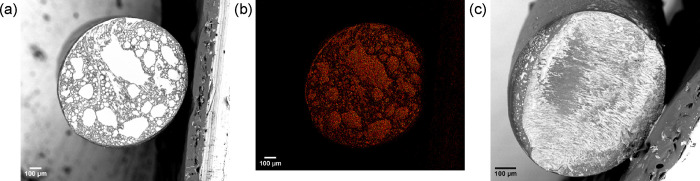
(a) Backscattered electron images of the cross sections of TPU-IL20 with (b) its pseudocolored EDX map sum of fluor and sulfur and (c) TPU-IL50.

**Figure 4:**
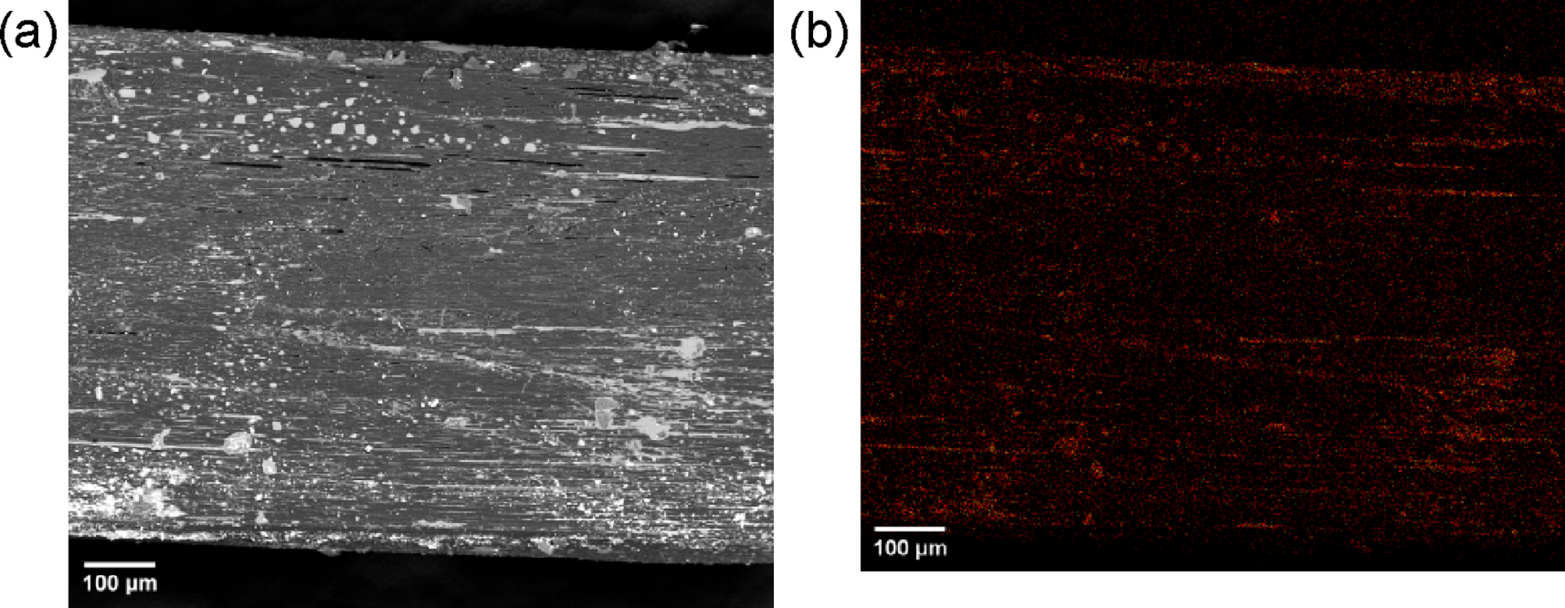
(a) Backscattered electron image of the longitudinal section of bulk ionofiber TPU-IL65­(MB) with (b) its pseudocolored EDX map sum of fluor and sulfur.

**Figure 5:**
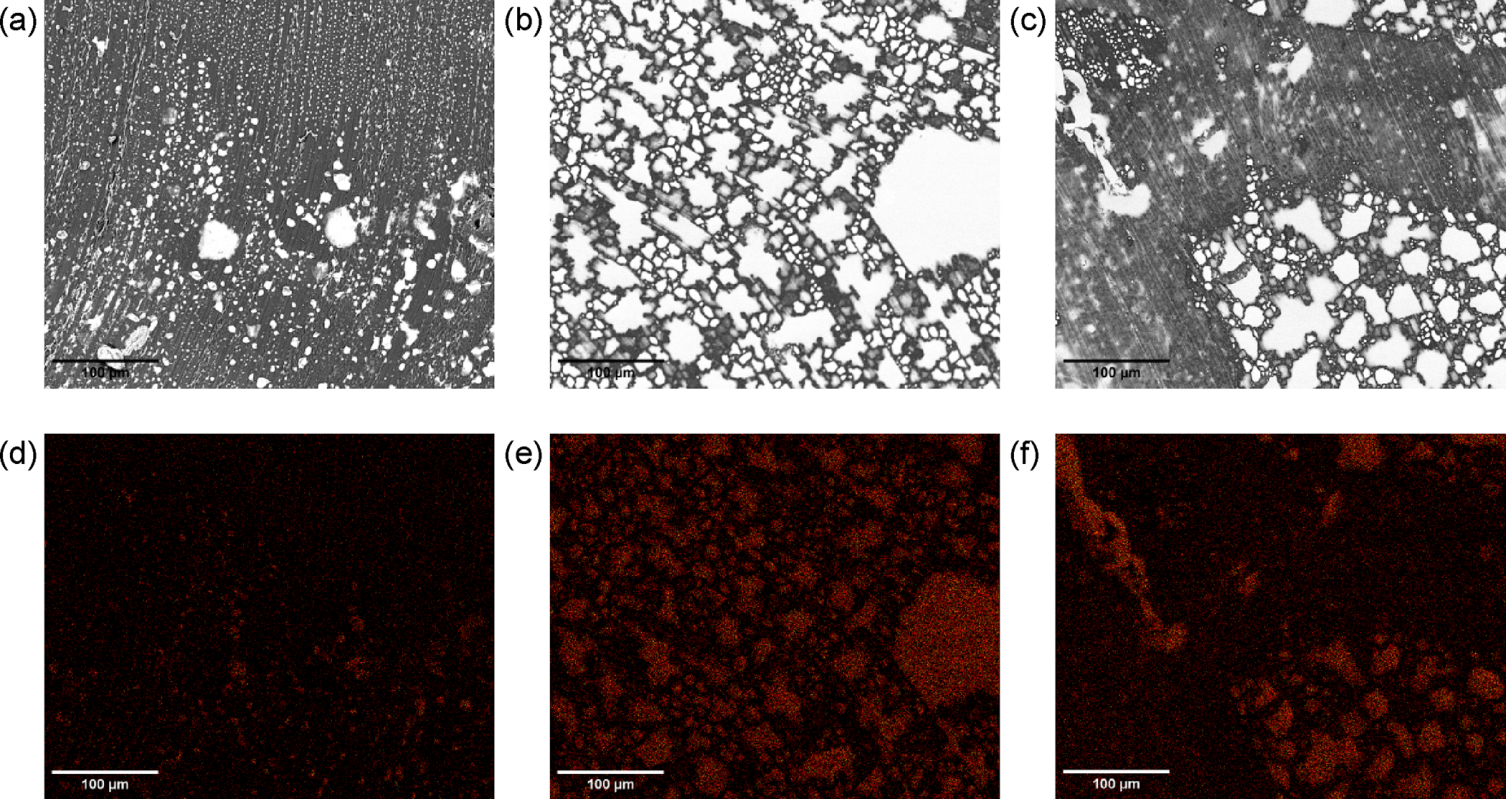
Close-up of backscattered electron images of the cross sections of the CNT-containing samples (a) TPU-CNT1.5-IL5, (b) TPU-CNT1.5-IL15, (c) TPU-CNT1.5-IL20, and (d–f) their respective pseudocolored EDX map sum of fluor and sulfur (same scale). Carbon EDX maps highlighting the segregated networks are available in the Supporting Information.

### 3.3 Monofilament Properties

The main properties of the produced monofilaments are illustratedin Figure 6a. As expected for TPU-based materials, the ultimate tensile properties were difficult to measure as every sample could be subject to considerable strain except for TPU-IL50, which broke relatively early at 110% strain (see the stress–strain curves in Figure 6b and Figure S8, Supporting Information). According to previous results, using ILs as ionically conductive medium tends to result to mechanically softer materials compared to their substrate.^1^ The pure bulk ionofibers were also observed to be softer, with their tensile modulus going from 31.6 mN tex^–1^ for vTPU to a range of 15.7 to 21.7 mN tex^–1^. Identically to vTPU, TPU-IL20 and TPU-IL65 showed over 1300% strain whereas TPU-IL35 would break at around 900% strain and TPU-IL50 at 100%. Our CPC monofilaments illustrated the effect of the CNT concentration on the increase of tensile modulus, as previously observed.^27^ Due to the faster crystallization rates induced with higher CNT concentrations, it was presumed that, after the extrudates were drawn, TPU chains aligned and consequently the tensile moduli increased. This was also seen for all the hybrid ionofibers as they had similar or higher tensile moduli than vTPU. However, compared to TPU-CNT1.5, the hybrid ionofibers TPU-CNT1.5-IL5 to TPU-CNT1.5-IL15 melt-spun at similar temperatures (210 °C) were indeed slightly softer.

**Figure 6:**
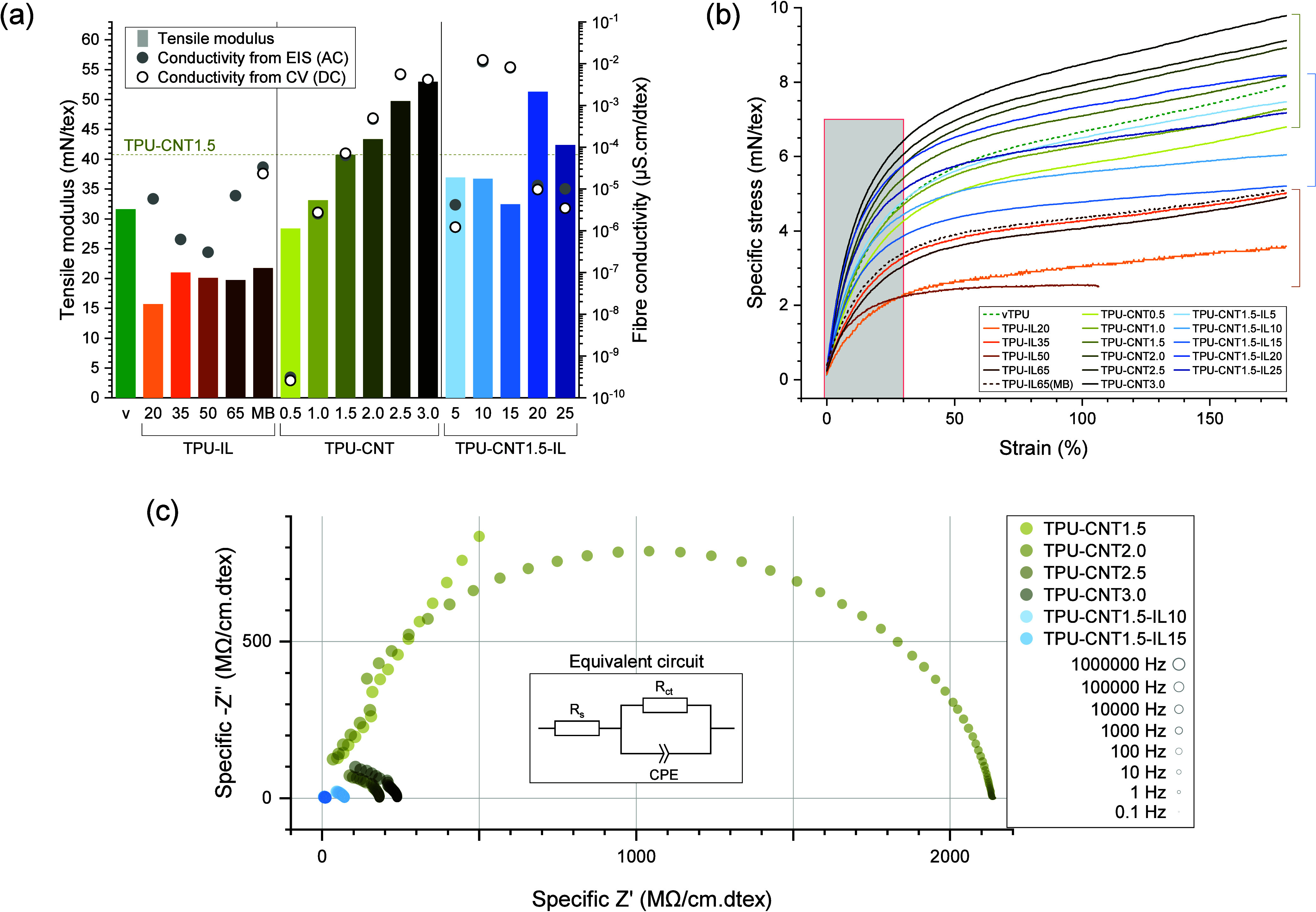
(a) Tensile moduli (columns) and fiber conductivities (points) of the samples. (b) Specific stress of the melt-spun samples up to 180% strain. 1 mN tex^–1^ is equivalent to 1.12 MPa for virgin TPU. A close-up of the first 30% strain is available in the Supporting Information. (c) Nyquist plot of the most conductive CNT-containing samples along with a typical equivalent circuit that was used for the fitting. The extracted *R*_ct_ was used for calculating the fiber conductivity.

Due to earlier measurements soon after their production, the pure ionofibers made from the pellets of the varied IL ratio batch had their fiber conductivity (σ_f_) calculated from their EIS results only. A direct correlation between the IL weight ratio and σ_f_ was not possible, probably due to the quality of the TPU-IL pellets. The TPU-IL35 sample, on which EIS was performed 9 days after being produced, was kept uncovered on a shelf in the laboratory for a year (with fluctuating temperature and relative humidity observed between 19 and 24 °C and 10–71%RH) and then remeasured in similar conditions. The resulting resistance extracted from the EIS was 665.97 MΩ compared to 674.55 MΩ from a year before, thus manifesting no clear sign of aging from the measurement of the conductivity of the sample. The Nyquist plot including the most conductive samples is presented in Figure 6. Figure S9 in the Supporting Information shows the frequencies of the data points selected for the circle fittings matching most of the charge-transfer semicircle. In some cases (TPU-CNT0.5, TPU-CNT2.5, TPU-CNT3.0, TPU-CNT1.5-IL10, and TPU-CNT1.5-IL15), σ_f_ was so low or so high that the Nyquist plot resulted in only the left or right half of the semicircle, respectively (all of the results from the EIS are available in Figures S10 and S11, Supporting Information). To some degree, σ_f_ calculated from EIS were close to those calculated from CV. At similar temperature and 27%RH, σ_f_ from the best of our pure bulk ionofibers, TPU-IL65­(MB), was in the same order of magnitude compared to that of surface ionofibers made with specifically designed ionogels containing the same IL (3.34 × 10^–5^ vs 3.87 × 10^–5^ μS cm dtex^–1^ at 10%RH).^1^ When only accounting for the apparent linear density of IL for each, this discrepancy of σ_f_ is three to four times bigger partly due to the heterogeneous sizes of IL-heavy phases: 5.13 × 10^–5^ vs 2.1 × 10^–4^ μS cm dtex^–1^.

Compared to their reference sample TPU-CNT1.5, the hybrid ionofibers showed either higher or lower σ_f_. For TPU-CNT1.5-IL10 and TPU-CNT1.5-IL15, σ_f_ reached higher than any of the CPC monofilament, with 1.12 × 10^–2^ μS cm dtex^–1^ from the EIS of the former. According to Fritzsche et al.,^19^ the increase of σ_f_ cannot be related to the IL itself and should be traced back to the CNT percolation network. With an optimum dispersion of the multiwalled CNTs between the IL-heavy achieved for these two samples, the more segregated conducting networks would explain the increase of σ_f_, as similarly seen on blends of TPU and imidazolium-based IL together with other carbon fillers.^17,20,26^ Due to using ion-blocking (gold-plated) electrodes for the measurements, it is unclear whether ionic pathways actually contribute to the transfer of charges. Nonetheless, it was observed on the other hybrid ionofibers that ion mobility was not out of the question (see, e.g., Figure S10c, Supporting Information). The charge-transfer semicircles were followed by a 45° line associated with diffusion processes (usually modeled with a Warburg element) that were characteristic of our ionofibers albeit more distinct on pure ones. Additionally, there was also observable charge storage on the CVs of some of our ionofibers (see Figure S12b, Supporting Information).

However, the most interesting observation on the hybrid ionofibers is the effect of concentration on the tunneling contribution to the ohmic conduction. Indeed, in percolated systems, it is believed that most of the conduction is ohmic in case the fillers are in close contact and tunneling transport when electrons need to hop from a nanofiller to the other by crossing an energy barrier.^28^ This tunneling contribution to the ohmic conduction is credited to the increase of tension applied (*V*) to the material and can be conveniently observed via the resulting current (*I*) through the *n*-value in the allometric eq 2 being superior to 1.^29^

$$I=aV^{n}$$ 2

From the fitted *n*-values of eq 2, it was quantifiable how much the concentration of CNTs influenced the tunneling contribution (Figure 7). This contribution was most present at percolation, as seen with TPU-CNT1.5. However, when the IL was also part of the system, the tunneling contribution was strongly attenuated, especially for TPU-CNT1.5-IL10 and TPU-CNT1.5-IL15. This was again presumed as the result of the more segregated conducting network.

**Figure 7:**
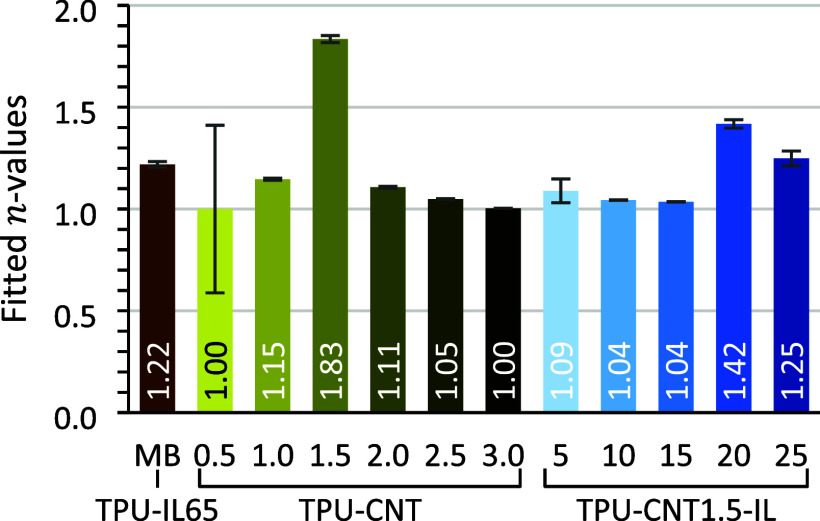
Fitted *n*-values for allometric scaling of the cyclic voltammetries. Values further away from 1 (ohmic conduction) represent bigger tunneling contributions.

### 3.4 Knittability

Using the monofilaments that were produced in bigger quantities (i.e., TPU-IL20 and TPU-CNT1.5-IL5), their textile processability was tested through knitting. The knitting of a 1 × 1 rib structure was performed without difficulties (Figure 8). The only minor issues encountered during the knitting process were due to the variation of the linear density of the monofilament. However, the elastomeric properties of TPU allow for a broad window of processability. The higher stiffness of TPU-CNT1.5-IL5 was also worth noting.

**Figure 8:**
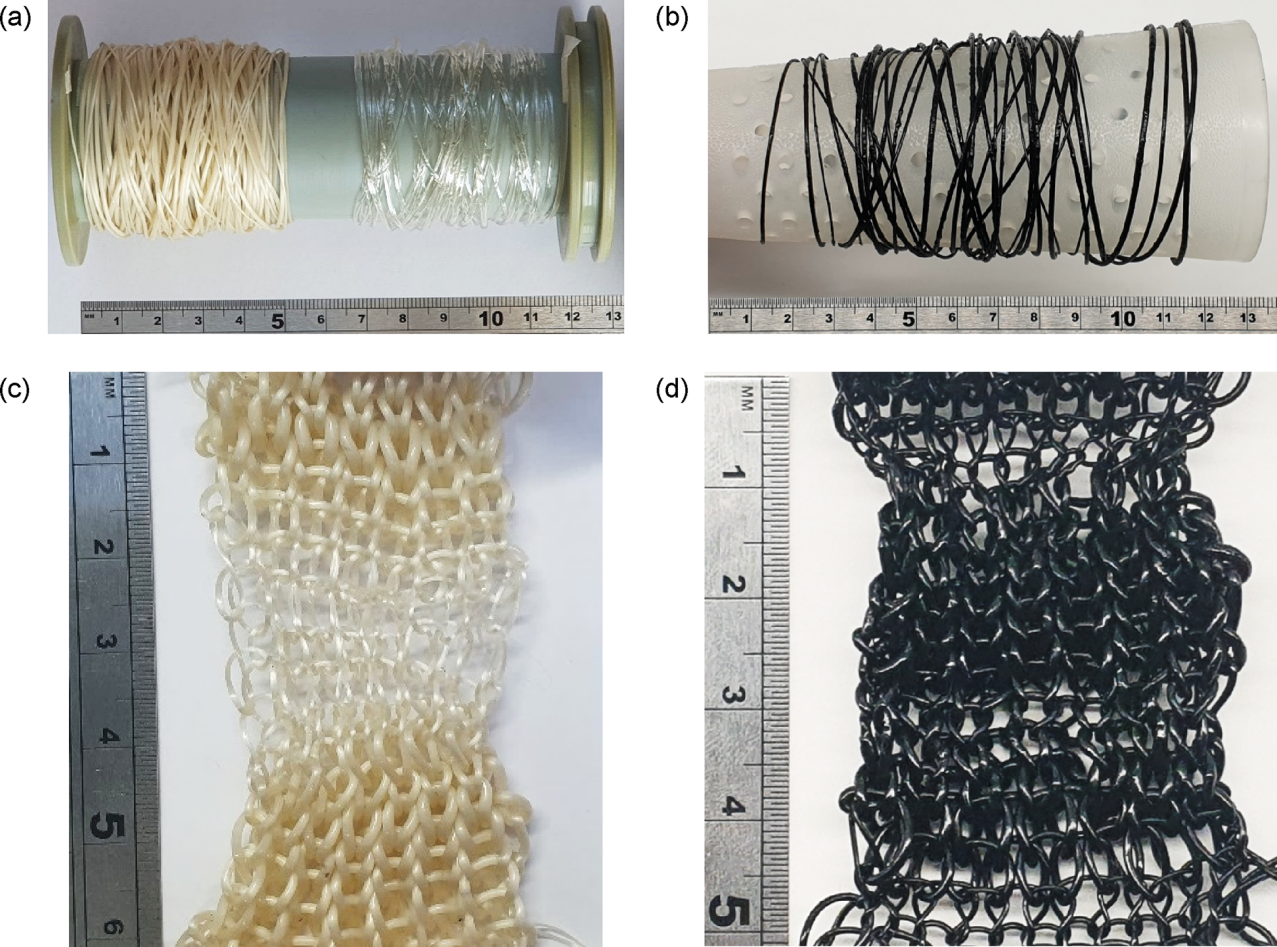
Melt-spun monofilaments (a) TPU-IL20, vTPU, and (b) TPU-CNT1.5-IL5, and the knitted fabrics made using (c) TPU-IL20 and (d) TPU-CNT1.5-IL5.

## 4 Conclusions

The melt-spinning process was investigated thoroughly as a method to produce bulk ionofibers based on TPU. This includes pure ionofibers using only an IL as ionically conductive medium but also hybrid ionofibers containing additionally multiwalled CNTs. The pure bulk ionofibers reached fiber conductivities similar to surface ionofibers up to the range of 10^–5^ μS cm dtex^–1^ (analogous to 10^–5^ S cm^–1^) at 27%RH, while containing a higher weight ratio of IL. There are approaches that can enhance said conductivity, e.g. by introducing ion channels.^30^ Bulk ionofibers have the advantage of being massive ion-sink/-source in comparison to surface ionofibers. Regarding the hybrid ionofibers, the IL concentration was observed to influence the morphology on the monofilaments and consequently of the properties of the produced monofilaments. An optimal window of IL concentration was identified to form segregated conducting networks of CNTs. This resulted in fiber conductivities within the range of 10^–2^ μS cm dtex^–1^, which was above that of monofilaments with concentrations past the percolation threshold of CNTs with no IL. However, since the electrochemical processes are inherently convoluted, it requires additional electrodes (e.g., electron-blocking) for a clearer interpretation of the results.^31^ Moreover, the potential toxicity and environmental persistence of an IL as ionically conductive medium (as well as other chemicals used) must be considered before further upscaling.^32^ The selection of the TPU grade should also be considered in the design of the TPU-based bulk ionofibers to ensure a good compatibility with the IL and the production process.

Nonetheless, melt spinning is an appropriate and flexible process for producing the fibers with different sets of properties (cross-sectional shape, number of monofilaments, drawing ratio, etc.). With the current set of properties, it was already possible to knit the fibers into a fabric using a manual machine. As a fiber-forming process, melt-spinning can answer the need of ionofibers for the manufacturing of ionotronic fabrics with unique functions and characteristics.

## Supplementary Material



## Notes

Additional information on the extrusion temperatures, the thermal analysis of the parallelly prepared TPU-IL65­(MB) pellets, the electrochemical spectroscopy, and cyclic voltammograms, including additional scanning electron microscopy images and energy-dispersive X-ray maps, and enlarged views from the differential scanning calorimetry and the stress–strain curves (DOCX)
