# Correction to “Melt Spinning of Thermoplastic Polyurethane-Based Bulk Ionofibers Filled with Carbon Nanotubes”

**DOI:** 10.1021/acsapm.5c02442

**Published:** 2025-07-23

**Authors:** Claude Huniade, Aurélie Cayla, Tariq Bashir, Nils-Krister Persson

In the original version of this article, Figure 2a and its related enlarged view Figure S1b in the Supporting Information are missing cooling data points from the differential scanning calorimetry (DSC) for sample TPU-IL20 and therefore the information related to its crystallization peak. The corrected Figure 2 is below, where the omitted cooling data points for TPU-IL20 are included. The corrected Figure S1b is in the associated Supporting Information, which includes the omitted cooling data points for TPU-IL20. These corrections do not alter the conclusion of this work.

**Figure 2:**
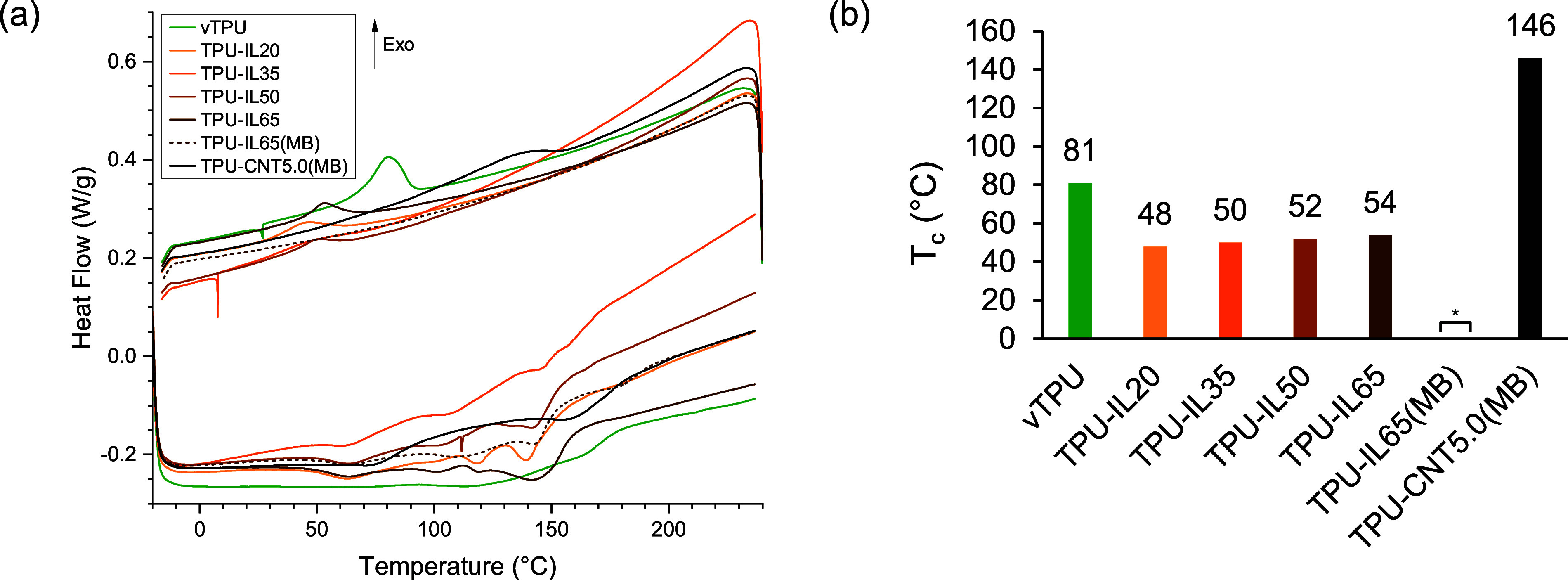
(a) DSC curves of prepared pellets. Additional plots with the enlarged view of the endothermic peaks, the crystallization peaks, and with the full range of TPU-IL65­(MB) are available in the Supporting Information. (b) Extracted crystallization temperatures from peaks. *: No noticeable peak.

## Supplementary Material



## Notes

Additional information on the extrusion temperatures, the thermal analysis of the parallelly prepared TPU-IL65­(MB) pellets, the electrochemical spectroscopy, and cyclic voltammograms, including additional scanning electron microscopy images and energy-dispersive X-ray maps, and enlarged views from the differential scanning calorimetry and the stress–strain curves (PDF)

