# Supplementary material for: Melt Spinning of Thermoplastic Polyurethane-Based Bulk Ionofibers Filled with Carbon Nanotubes
Source: ACS Appl Polym Mater. 2025 May 23;7(11):6719–27. doi: 10.1021/acsapm.5c00286 (PMC12172015; doi:10.1021/acsapm.5c00286)
Supplement: Supplementary file 1 [file ap5c00286_si_001.docx]

Supporting Information

Melt-spinning of Thermoplastic Polyurethane-Based Bulk Ionofibers Filled with Carbon Nanotubes

Claude Huniade^1^*, Aurélie Cayla^2^, Tariq Bashir^1^, Nils-Krister Persson^1^

^1^Polymeric E-textiles, The Swedish School of Textiles, University of Borås, Borås 501 90, Sweden

^2^University of Lille, ENSAIT, ULR 2461 - GEMTEX - Génie et Matériaux Textiles, Lille 59000, France

*Email: claude.huniade@hb.se

Extrusion temperatures

**Table S1.** Temperature for barrel heating zones of the twin-screw extruder for the TPU-CNT masterbatch in order. The main feed zone Z1 is permanently cooled without temperature control.

| Barrel heating zones | Z2 | Z3 | Z4 | Z5 | Z6 | Z7 | Z8 | Die |
| --- | --- | --- | --- | --- | --- | --- | --- | --- |
| Temperature (°C) | 180 | 210 | 215 | 220 | 225 | 225 | 230 | 230 |

**Table** **S2.** Weight ratios of the different attempts of extrusion.
MB stands for masterbatch which has been used for the CNT containing attempts.

| Sample | TPU wt% | CNT wt% | IL wt% |
| --- | --- | --- | --- |
| vTPU | 100.0 | − | − |
| TPU-IL20 | 80.0 | − | 20.0 |
| TPU-IL35 | 65.0 | − | 35.0 |
| TPU-IL50 | 50.0 | − | 50.0 |
| TPU-IL65 | 35.0 | − | 65.0 |
| TPU-IL65(MB) | 35.0 | − | 65.0 |
| TPU-CNT5.0(MB) | 95.0 | 5.0 | − |
| TPU-CNT0.5 | 99.5 | 0.5 | − |
| TPU-CNT1.0 | 99.0 | 1.0 | − |
| TPU-CNT1.5 | 98.5 | 1.5 | − |
| TPU-CNT2.0 | 98.0 | 2.0 | − |
| TPU-CNT2.5 | 97.5 | 2.5 | − |
| TPU-CNT3.0 | 97.0 | 3.0 | − |
| TPU-CNT1.5-IL5 | 93.5 | 1.5 | 5.0 |
| TPU-CNT1.5-IL10 | 88.5 | 1.5 | 10.0 |
| TPU-CNT1.5-IL15 | 83.5 | 1.5 | 15.0 |
| TPU-CNT1.5-IL20 | 78.5 | 1.5 | 20.0 |
| TPU-CNT1.5-IL25 | 73.5 | 1.5 | 25.0 |
| TPU-CNT1.5-IL30 | 68.5 | 1.5 | 30.0 |
| TPU-CNT1.5-IL35 | 63.5 | 1.5 | 35.0 |
| TPU-CNT1.5-IL40 | 58.5 | 1.5 | 40.0 |

**Table S3.** Extrusion temperature for the melt-spinning of the drawn and nondrawn extrudates.

| Drawn extrudates (1.5 m min^−1^) | Temperature (°C) | Nondrawn extrudates | Temperature (°C) |
| --- | --- | --- | --- |
| vTPU | 215 |  |  |
| TPU-IL20 | 170 |  |  |
| TPU-IL35 | 157 |  |  |
| TPU-IL50 | 140 |  |  |
| TPU-IL65 | 170 |  |  |
| TPU-IL65(MB) | 155 |  |  |
| TPU-CNT0.5 to 3.0 | 210 |  |  |
| TPU-CNT1.5-IL5 | 200 |  |  |
| TPU-CNT1.5-IL10 | 210 |  |  |
| TPU-CNT1.5-IL15 | 210 |  |  |
| TPU-CNT1.5-IL20 | 180 | TPU-CNT1.5-IL20(ND) | 180 |
| TPU-CNT1.5-IL25 | 180 | TPU-CNT1.5-IL25(ND) | 180 |
|  |  | TPU-CNT1.5-IL30(ND) | 170 |
|  |  | TPU-CNT1.5-IL35(ND) | 170 |
|  |  | TPU-CNT1.5-IL40(ND) | 170 |

Enlarged views from the differential scanning calorimetry (DSC)

| (a) |  | (b) |  |
| --- | --- | --- | --- |

**Figure S1.** Enlarged views on the DSC curves of (a) the endothermic peaks during heating and (b) the crystallization peaks during cooling.

**Figure S2.** Enlarged views on the DSC curve of virgin TPU
with indication of the endothermic events during heating.

Thermal analysis of the parallelly prepared TPU-IL65(MB) pellets

| (a) |  | (b) |  |
| --- | --- | --- | --- |

**Figure S3.** Results from (a) TGA and (b) DSC of eight parallelly prepared TPU‑IL65(MB) pellets. For the peak analysis of the DSC curve, only the dashed curve was used for the peak analysis.

Scanning electron microscopy (SEM) images and energy dispersive X-ray (EDX) maps

| (a) | 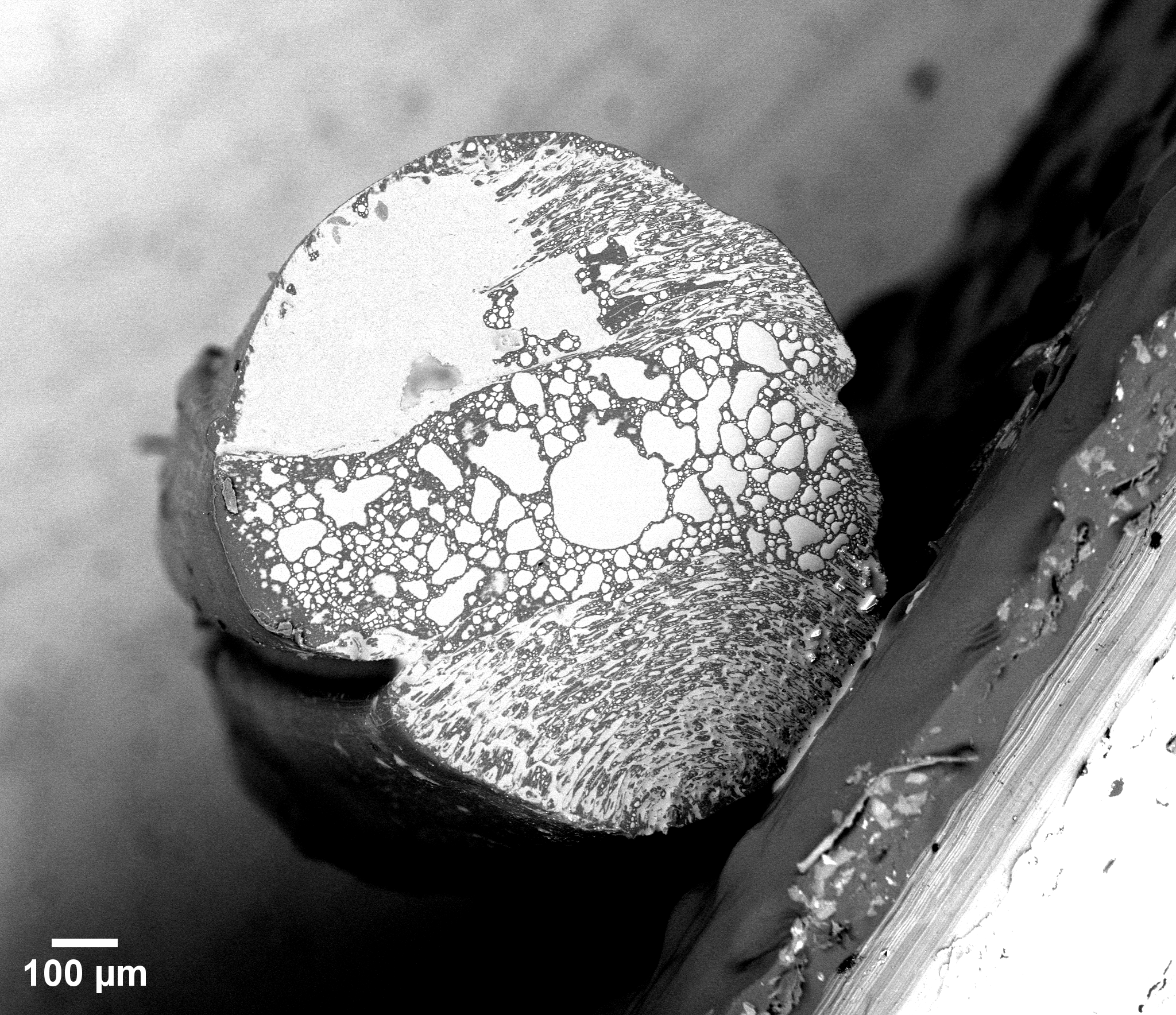 | (b) | 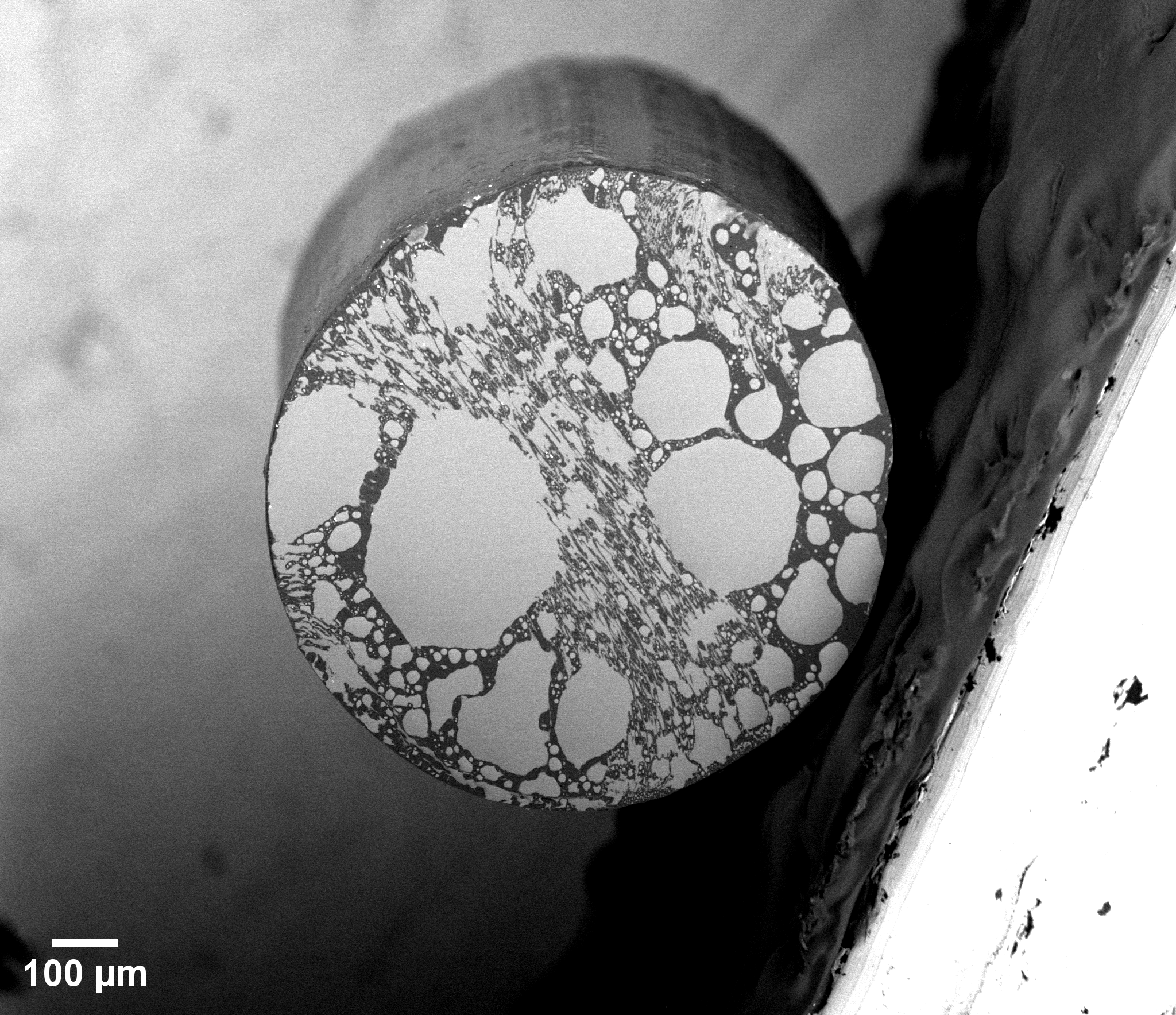 | (c) | 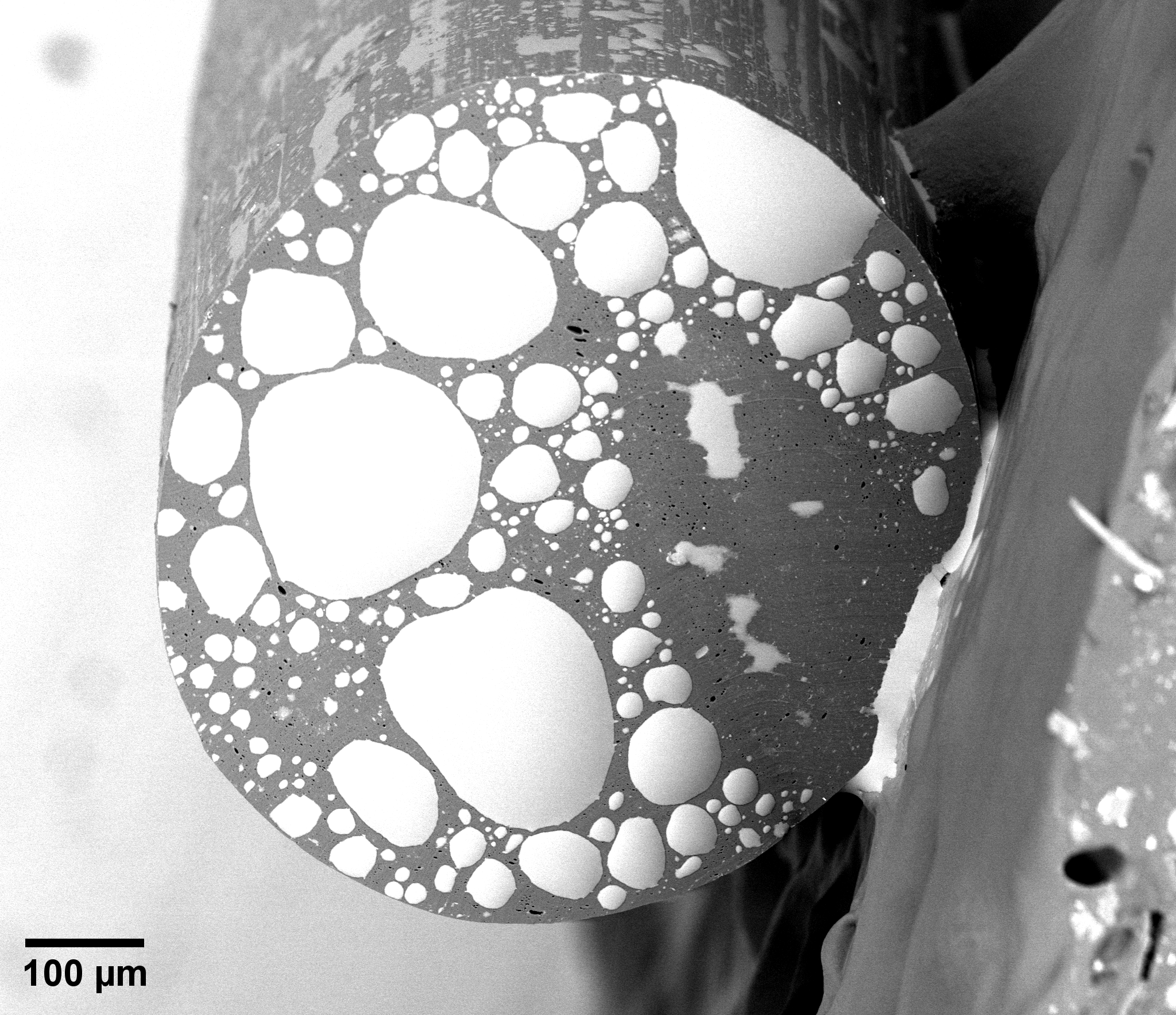 |
| --- | --- | --- | --- | --- | --- |

**Figure S4.** (a) Back-scattered electron images of the cross-sections of TPU-IL35,
(b) TPU-IL65, and (c) TPU-IL65(MB).

| (a) | 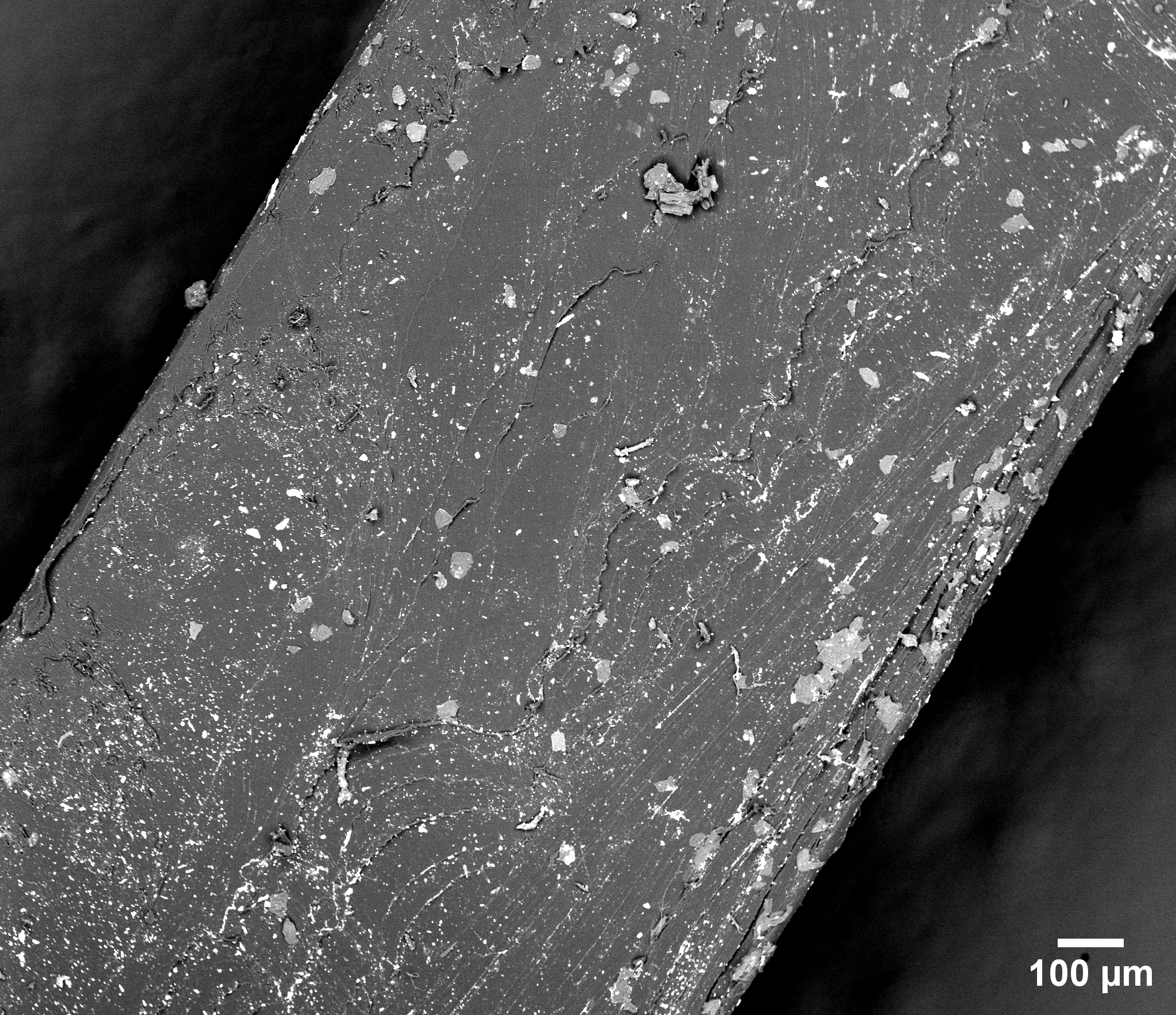 | (b) | 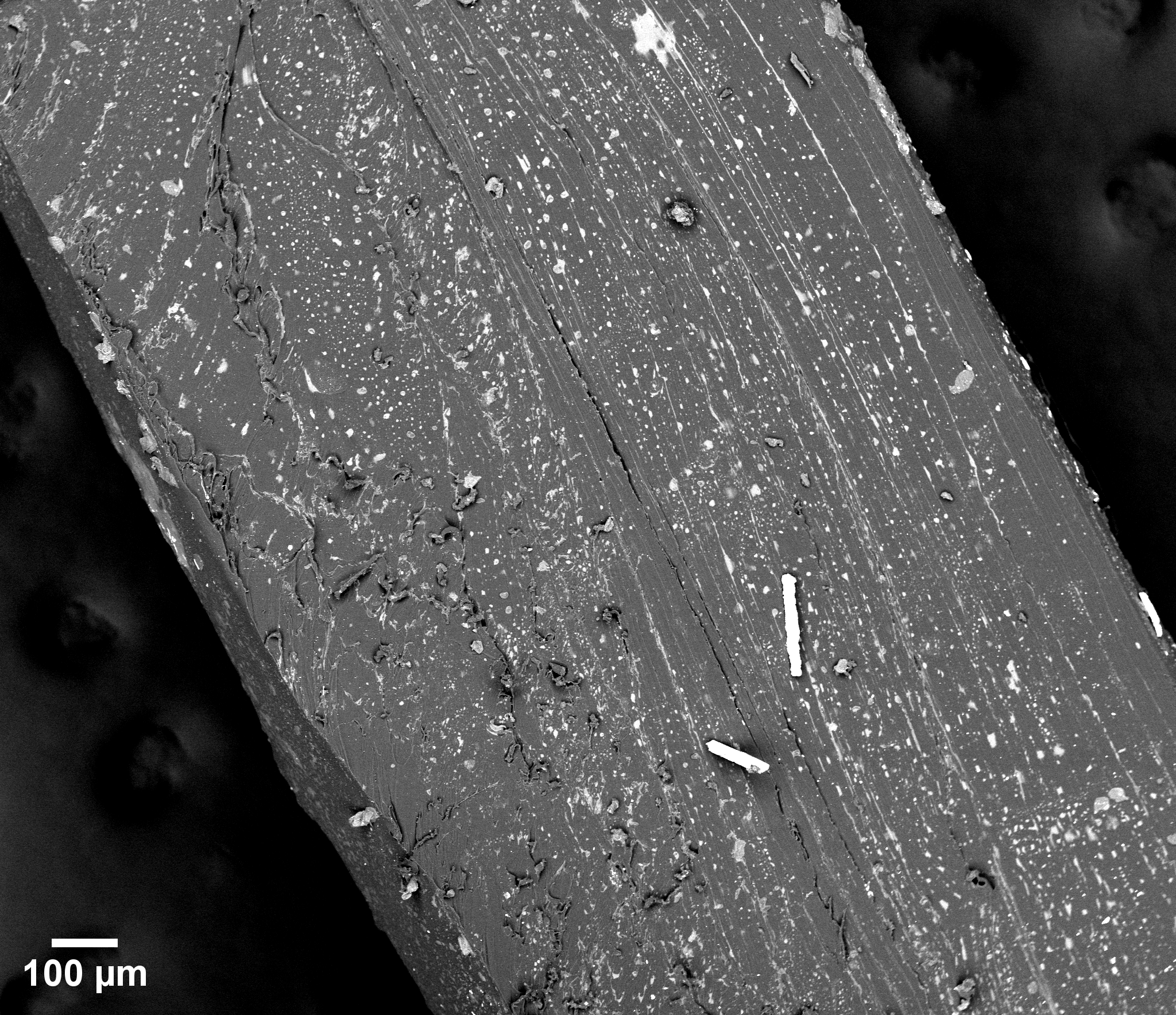 | (c) | 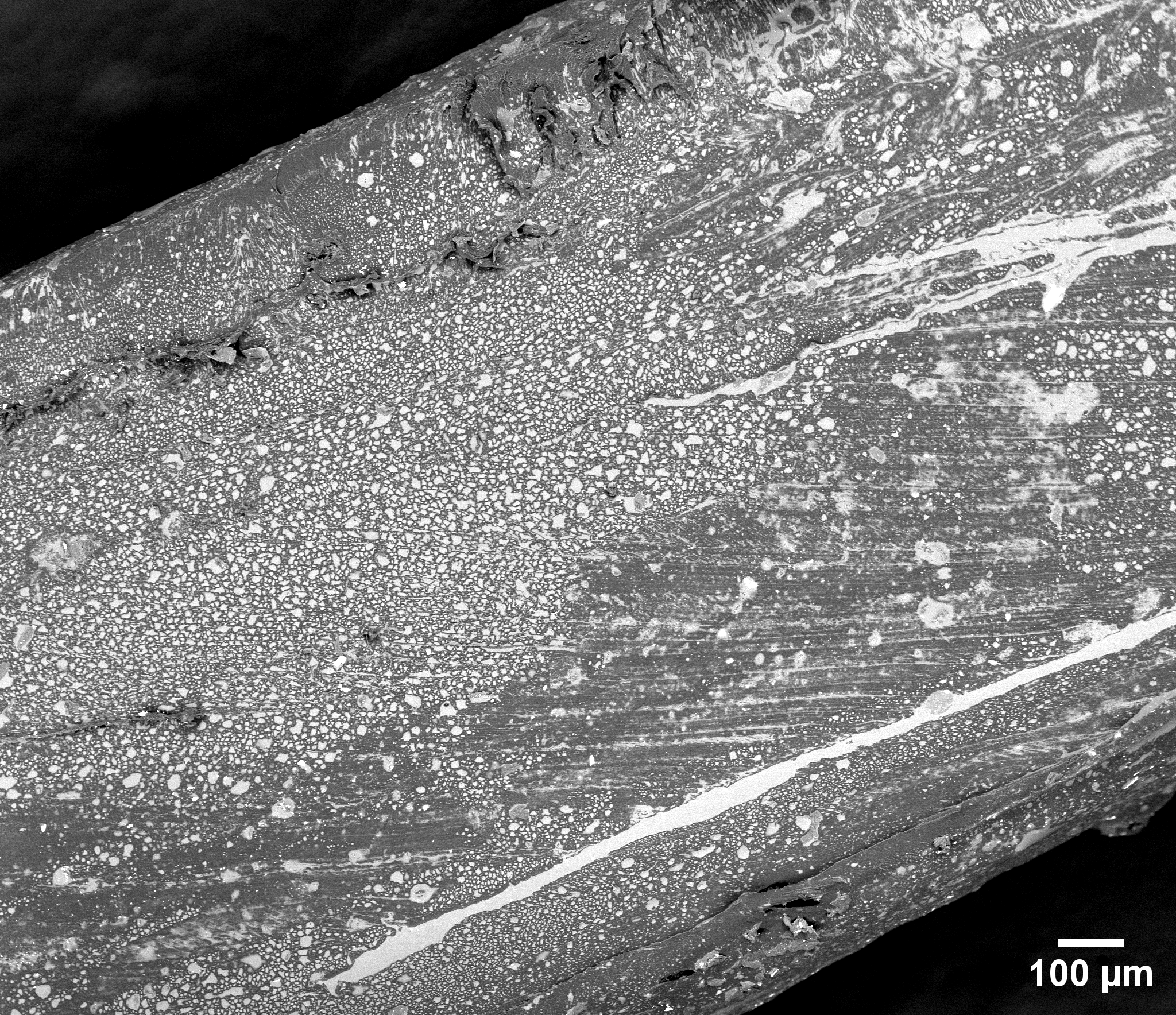 |
| --- | --- | --- | --- | --- | --- |
| (d) | 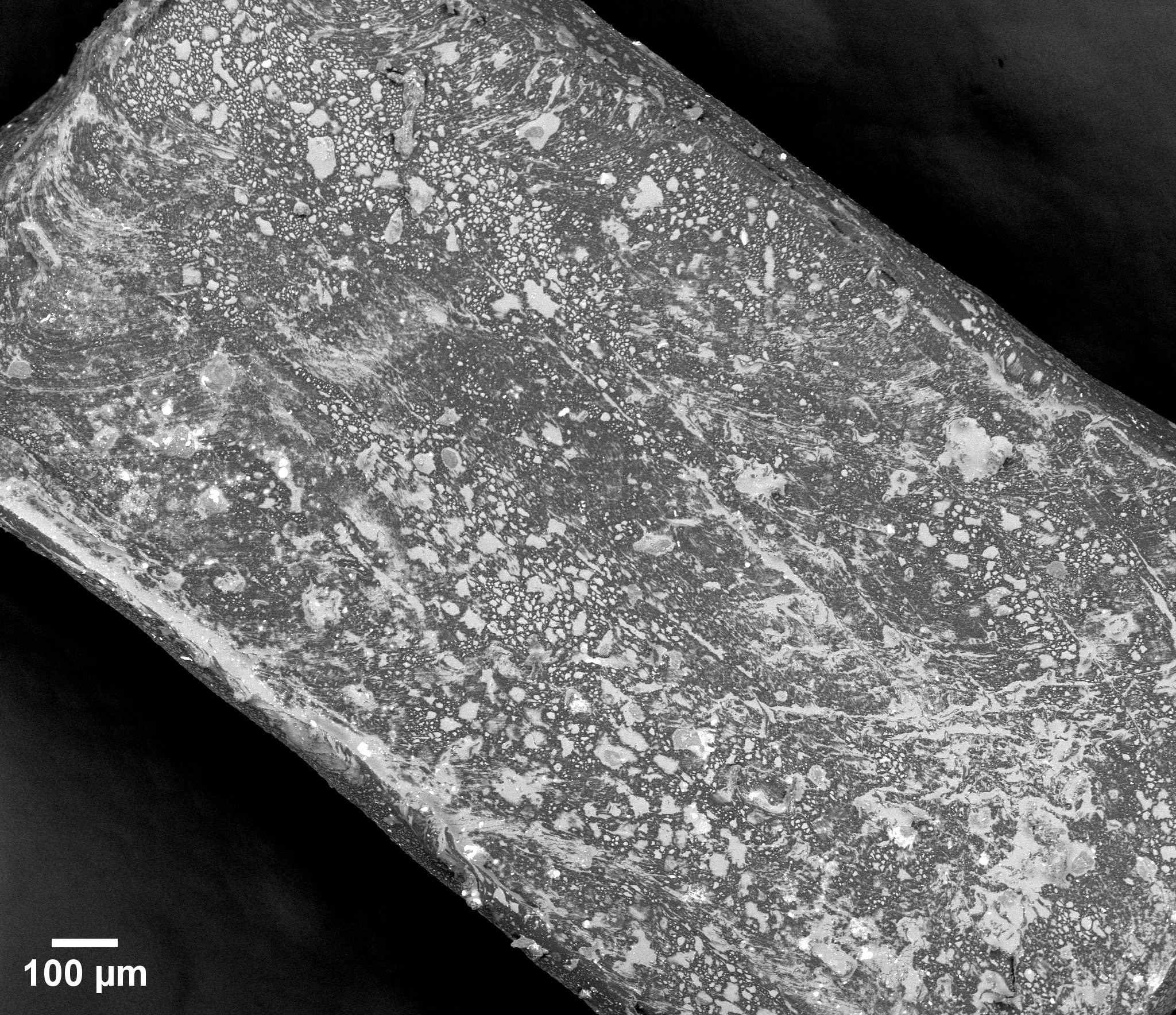 | (e) | 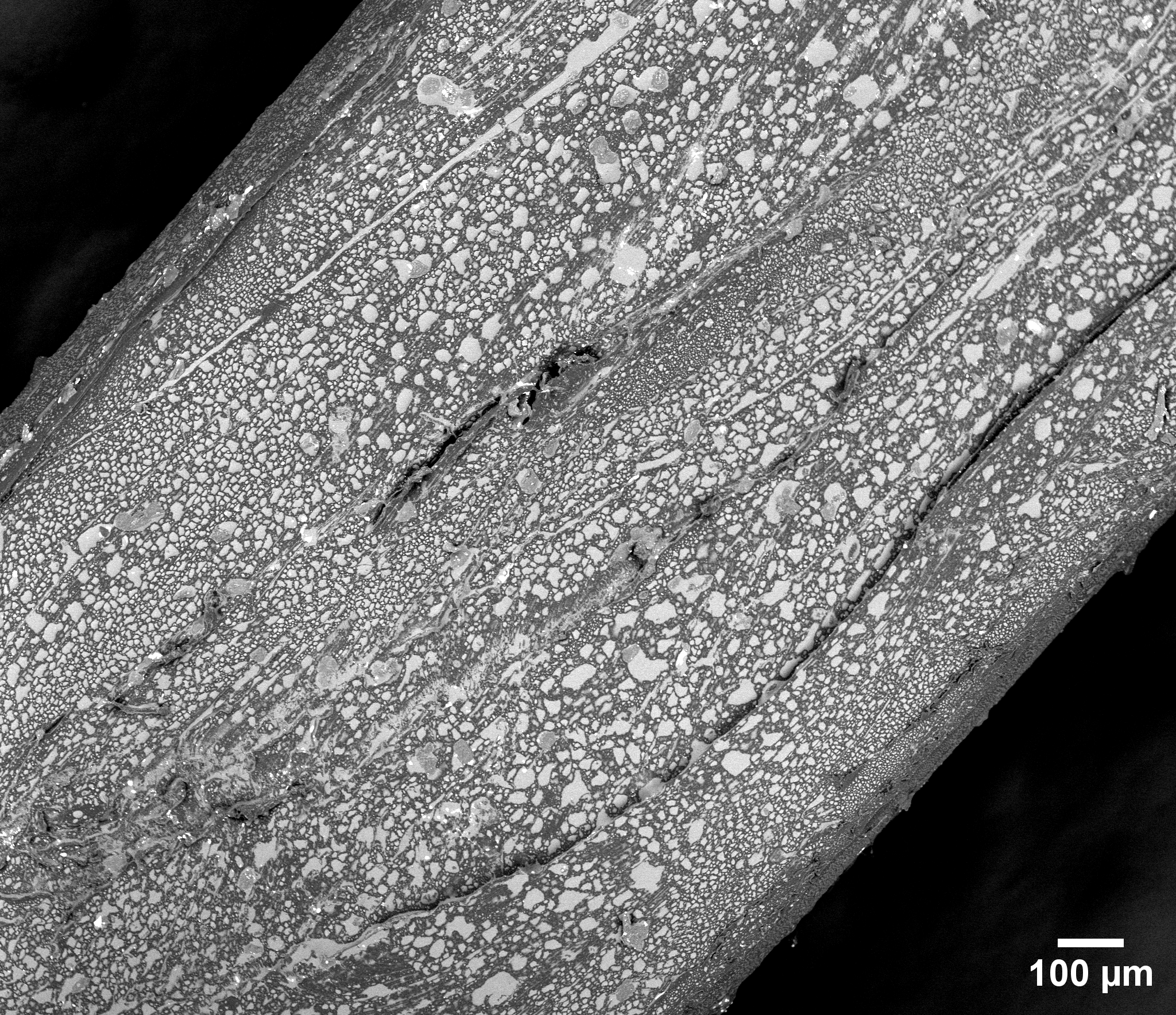 | (f) | 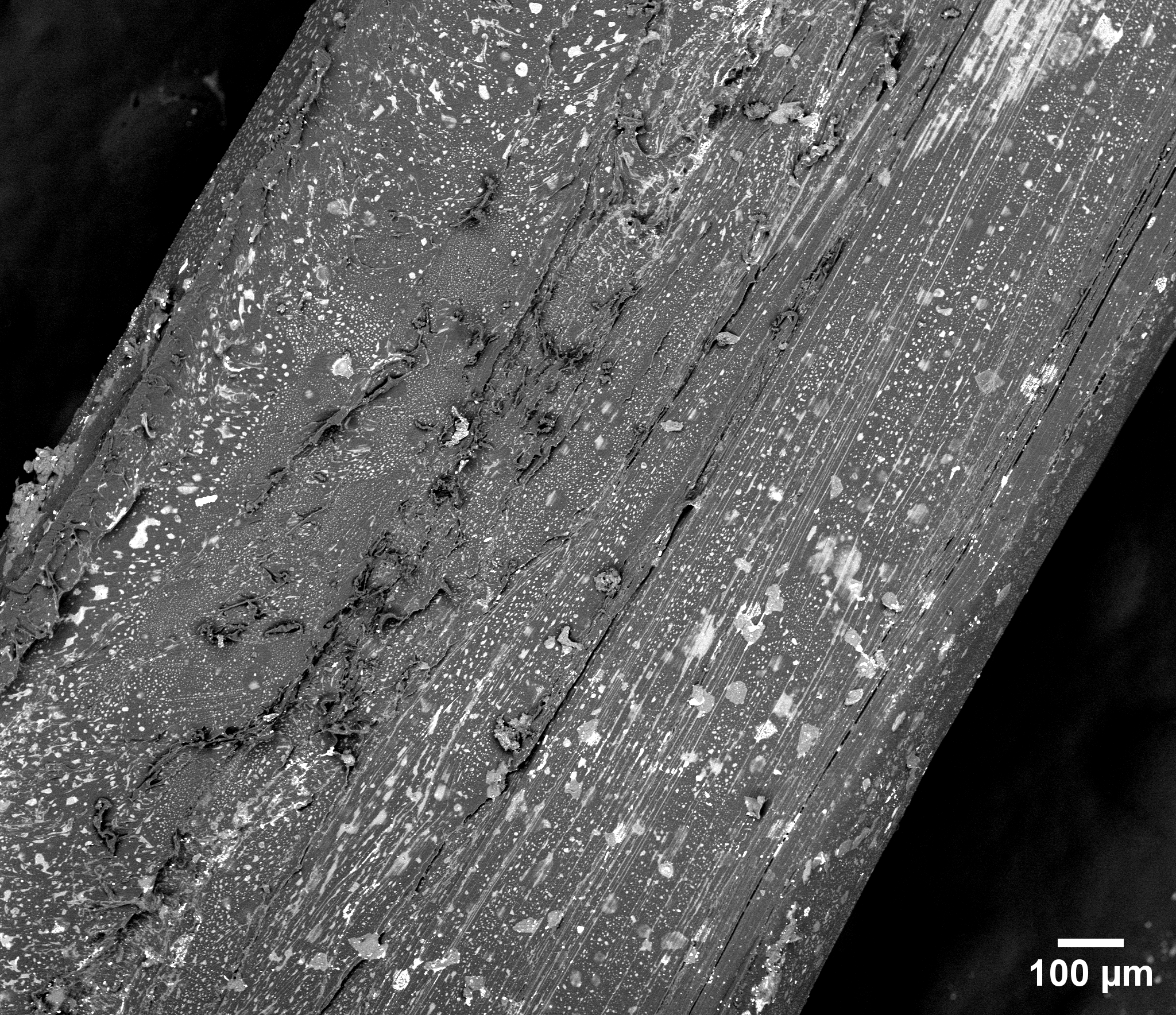 |

**Figure S5.** Back-scattered electron images of the longitudinal sections of the
CNT-containing samples (a) TPU-CNT1.5, (b) TPU-CNT1.5-IL5, (c) TPU-CNT1.5-IL10,
(d) TPU-CNT1.5-IL15, (e) TPU-CNT1.5-IL20, and (f) TPU-CNT1.5-IL25.

| (a) | 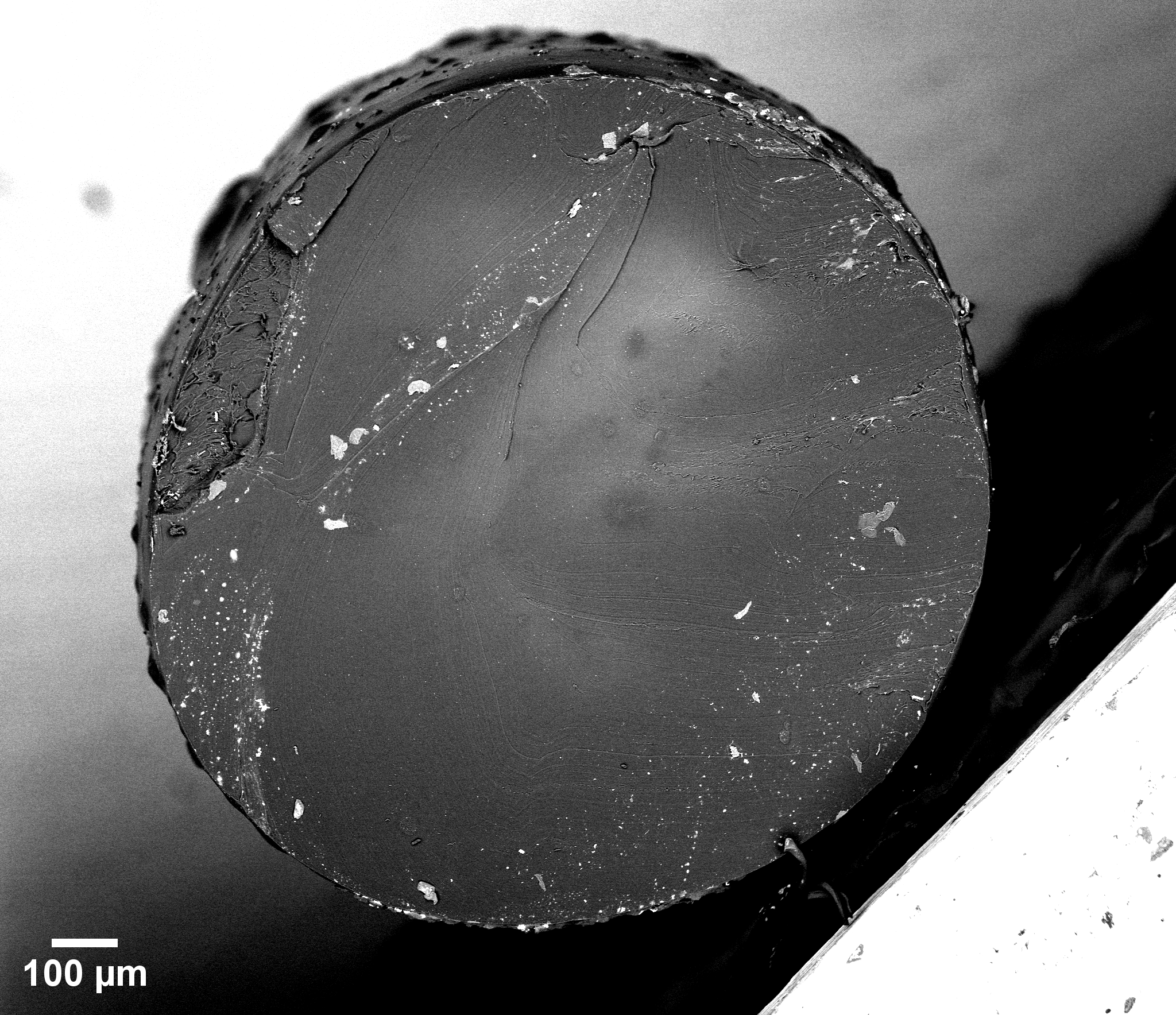 | (b) | 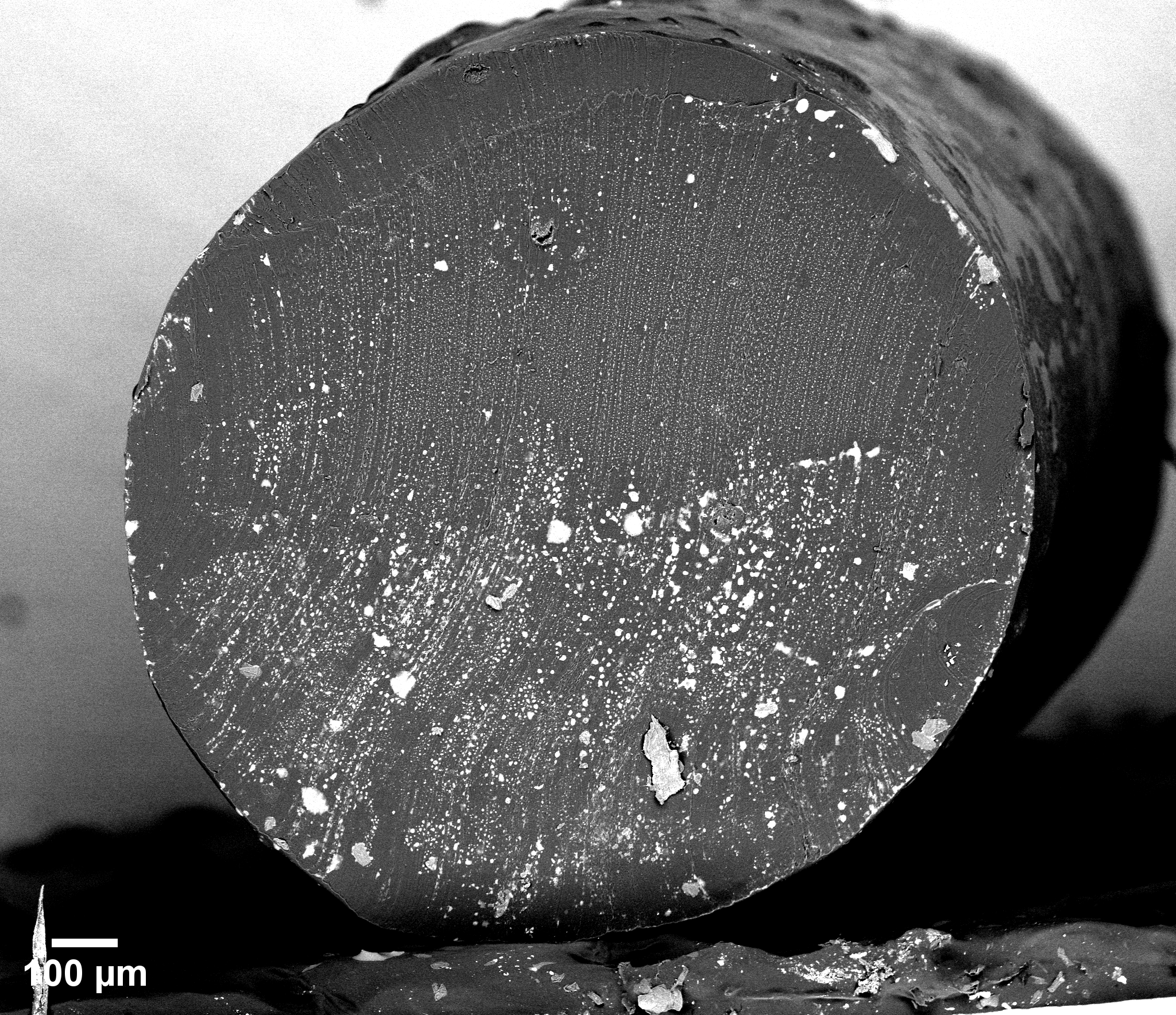 | (c) | 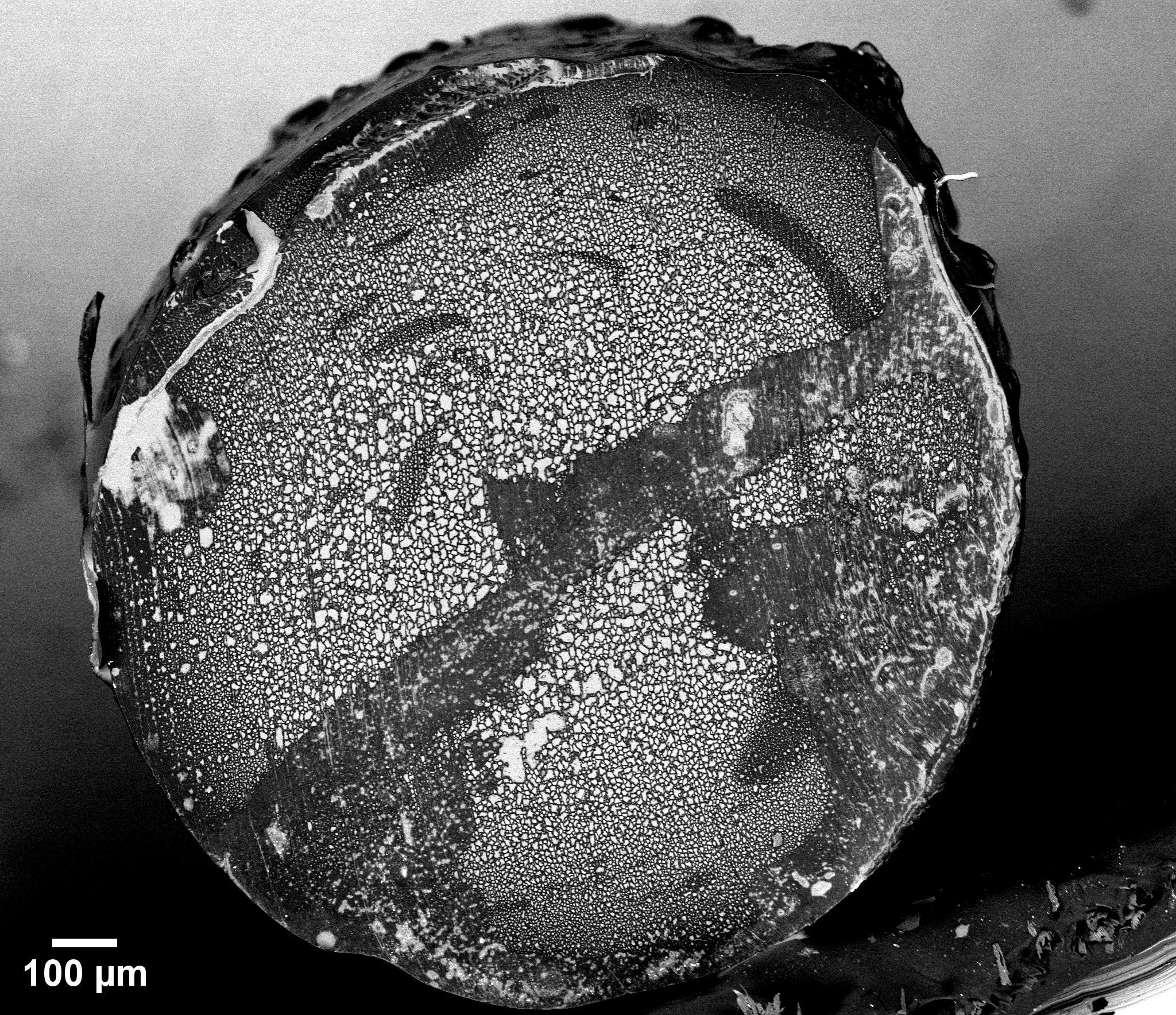 |
| --- | --- | --- | --- | --- | --- |
| (d) | 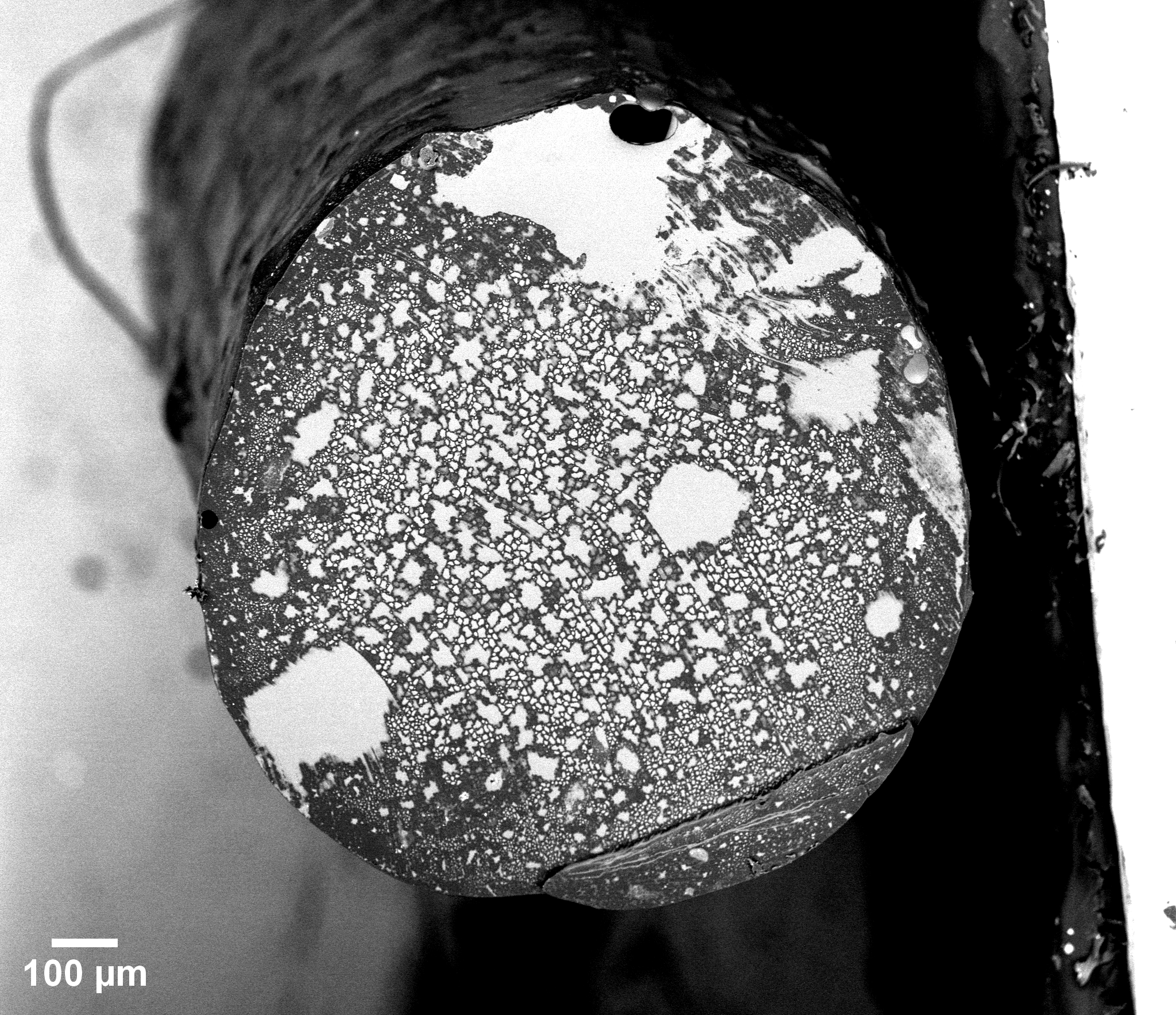 | (e) | 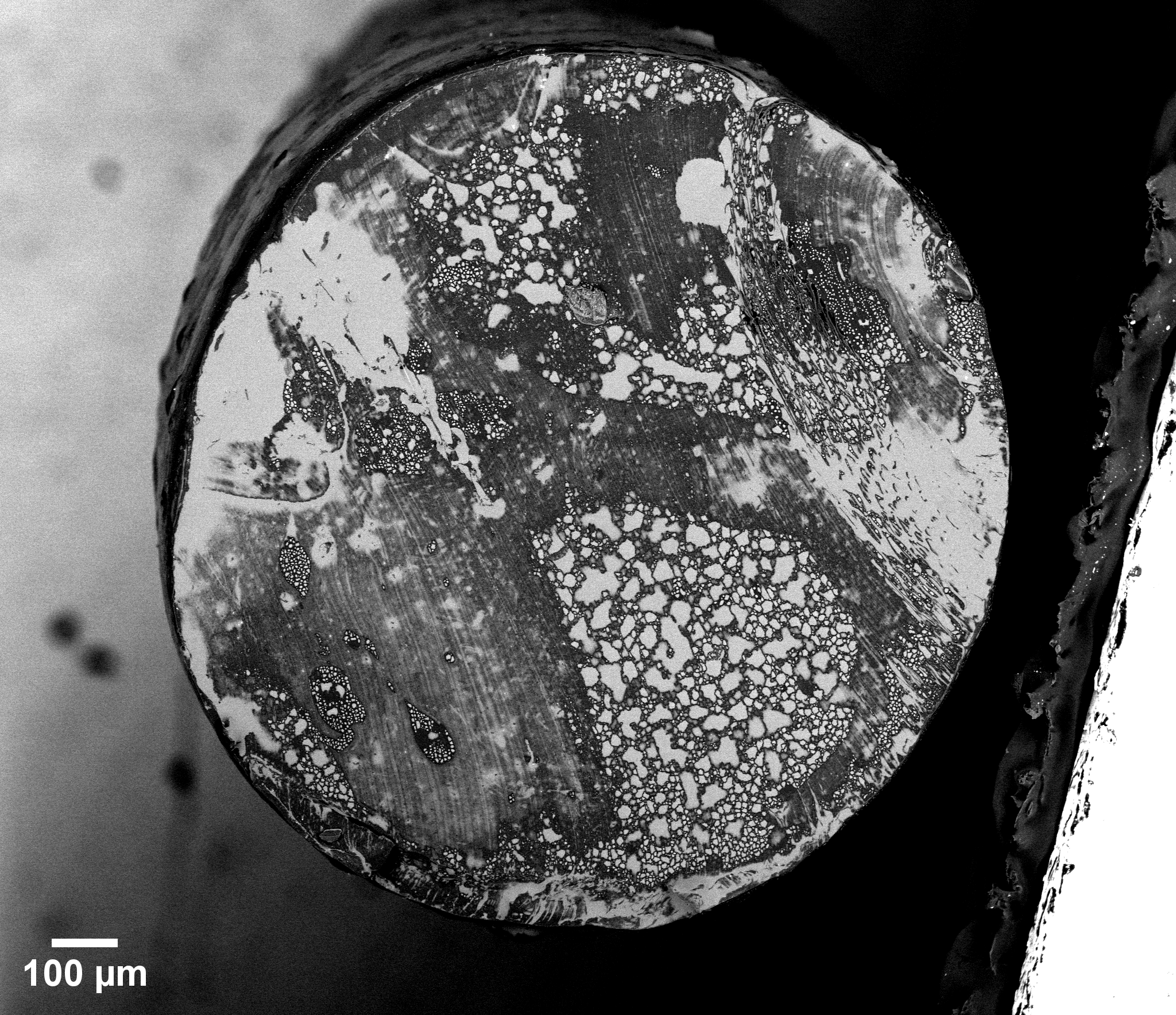 | (f) | 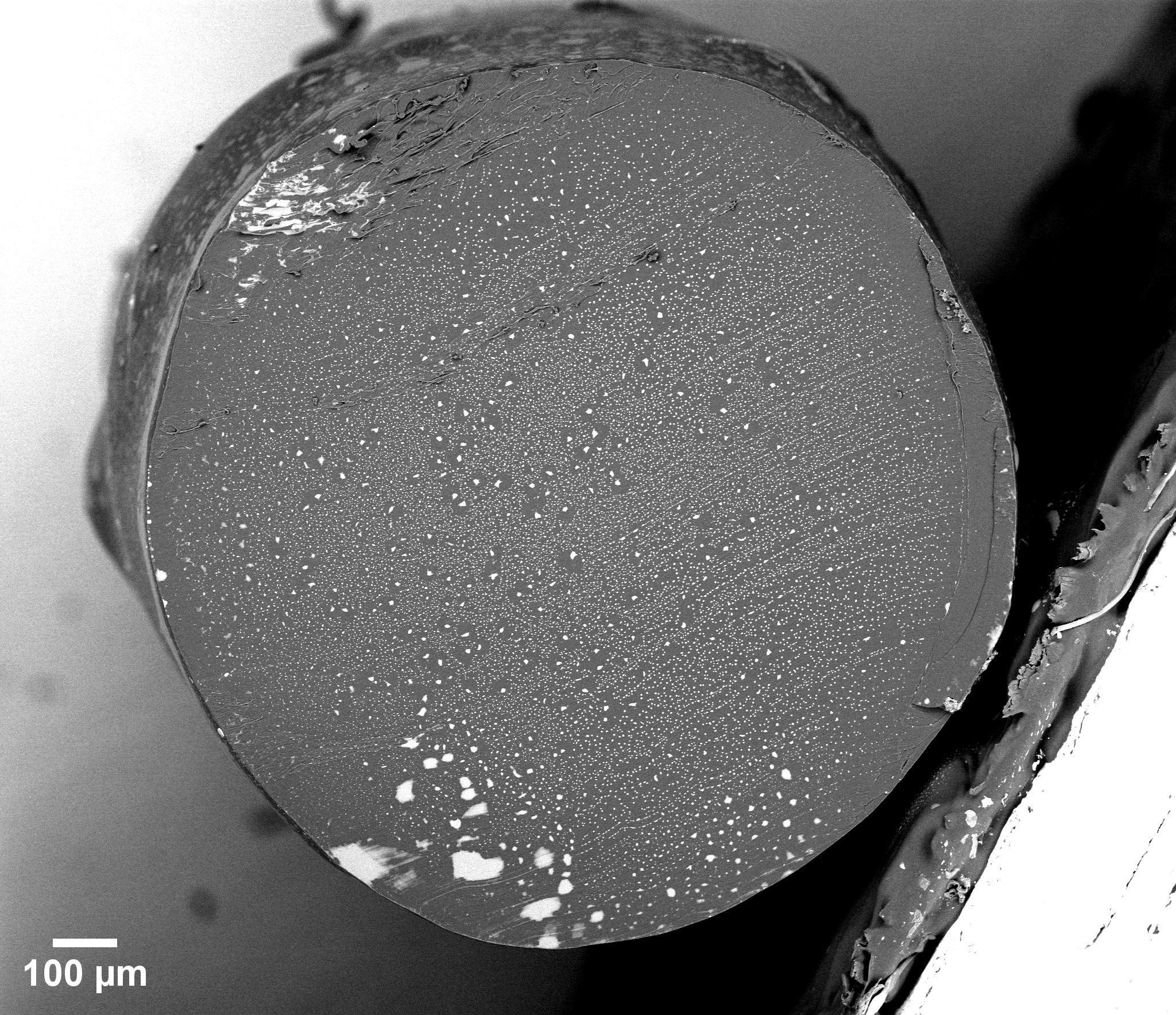 |

**Figure S6.** Back-scattered electron images of the cross-sections of the CNT-containing samples (a) TPU-CNT1.5, (b) TPU-CNT1.5-IL5, (c) TPU-CNT1.5-IL10,
(d) TPU-CNT1.5-IL15, (e) TPU-CNT1.5-IL20, and (f) TPU-CNT1.5-IL25.

| (a) | 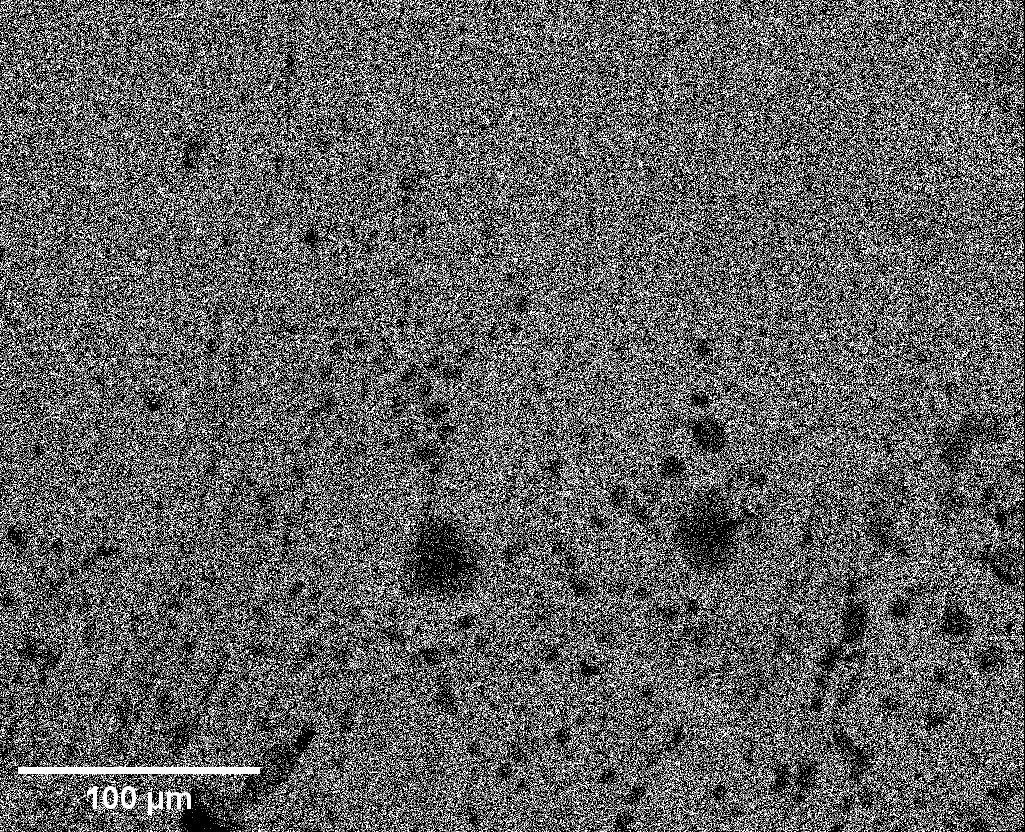 | (b) | 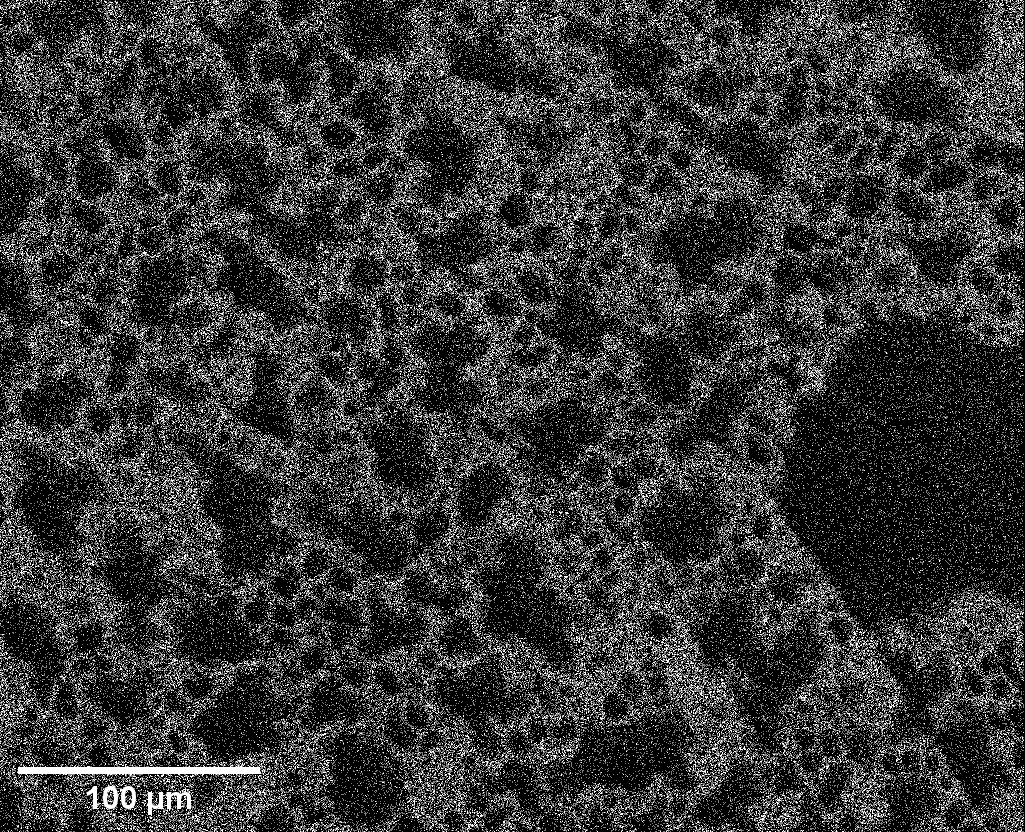 | (c) | 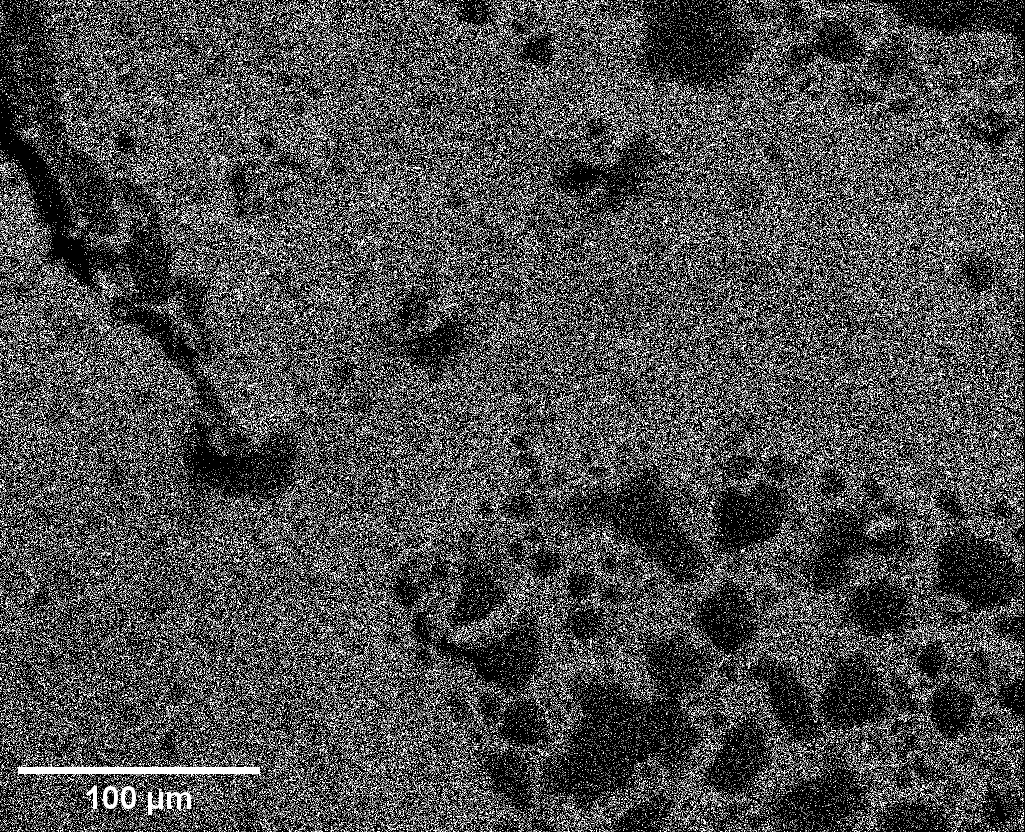 |
| --- | --- | --- | --- | --- | --- |

**Figure S7.** Close-up carbon EDX maps of the cross-sections of the CNT-containing samples
(a) TPU-CNT1.5-IL5, (b) TPU-CNT1.5-IL15, and (c) TPU-CNT1.5-IL20.
Brightness and contrast were increased to apply the following color pattern:
black for pixel value 0, grey for 1, white from 2 to 8.

Close-up of the stress-strain curves

**Figure S8.** Close-up of the stress-strain curves on the first 30 % of strain.
1 mN tex^−1^ is equivalent to 1.12 MPa for virgin TPU.

Additional information and plots from the electrochemical impedance spectroscopy.


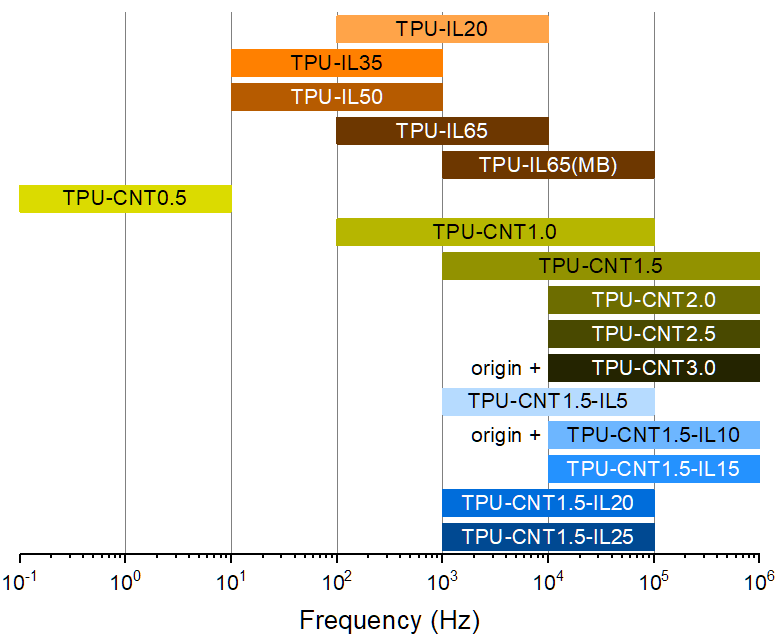


**Figure S9.** Range of frequencies selected for the semicircle fittings
in the Nyquist plots from the electrochemical impedance spectroscopy.
The origin was also added when it would otherwise result in an inaccurate fit.

| (a) |  |
| --- | --- |

| (b) |  |
| --- | --- |

| (c) |  |
| --- | --- |

| (d) |  |
| --- | --- |

**Figure S10.** Specific Nyquist plots with different scales around

(a) 10^9^, (b) 10^6^, (c) 10^5^, and (d) 10^4^ MΩ cm^−1^ dtex. (continued next page)

| (e) |  |
| --- | --- |

| (f) |  |
| --- | --- |

| (g) |  |
| --- | --- |

**Figure S10.** Specific Nyquist plots with different scales around

(e) 10^3^, (f) 10^2^, and (g) 10 MΩ cm^−1^ dtex. (continued from previous page)

| (a) |  |
| --- | --- |

| (b) |  |
| --- | --- |

| (c) |  |
| --- | --- |

**Figure S11.** Specific Bode plots at different scales, with the magnitude of the specific impedance up to (a) 10^10^, (b) 10^7^, and (c) 2×10^3^ MΩ cm^−1^ dtex.

Cyclic voltammograms from the cyclic voltammetry (CV)

| (a) |  | (b) |  |
| --- | --- | --- | --- |

| (c) |  | (d) |  |
| --- | --- | --- | --- |

| (e) |  |
| --- | --- |

**Figure S12.** Cyclic voltammograms with different scales around
(a) 10^−9^, (b) 10^−5^, (c) 10^−4^, (d) 10^−3^, and (e) 10^−2^ μA cm dtex^−1^.
