# Supplementary material for: Correction to “Melt Spinning of Thermoplastic Polyurethane-Based Bulk Ionofibers Filled with Carbon Nanotubes”
Source: ACS Appl Polym Mater. 2025 Jul 23;7(15):10326–7. doi: 10.1021/acsapm.5c02442 (PMC12340765; doi:10.1021/acsapm.5c02442)
Supplement: Supplementary file 1 [file ap5c02442_si_001.pdf]

Corrected Supporting Information

Melt Spinning of Thermoplastic Polyurethane-Based Bulk Ionofibers Filled with Carbon Nanotubes

*Claude Huniade[1] *, Aurélie Cayla[2], Tariq Bashir[1], Nils-Krister Persson[1]*

ACS Appl. Polym. Mater. 2025, 7, 11, 6719–6727. DOI: 10.1021/acsapm.5c00286

[1]Polymeric E-textiles, The Swedish School of Textiles, University of Borås, Borås 501 90, Sweden

[2]University of Lille, ENSAIT, ULR 2461 - GEMTEX - Génie et Matériaux Textiles, Lille 59000, France

*Email: claude.huniade@hb.se

Extrusion temperatures

Table S1. Temperature for barrel heating zones of the twin-screw extruder for the TPU-CNT masterbatch in order. The main feed zone Z1 is permanently cooled without temperature control.

Barrel heating zones	Z2	Z3	Z4	Z5	Z6	Z7	Z8	Die
Temperature (°C)	180	210	215	220	225	225	230	230

Table S2. Weight ratios of the different attempts of extrusion.
MB stands for masterbatch which has been used for the CNT containing attempts.

Sample	TPU wt%	CNT wt%	IL wt%
vTPU	100.0	–	–
TPU-IL20	80.0	–	20.0
TPU-IL35	65.0	–	35.0
TPU-IL50	50.0	–	50.0
TPU-IL65	35.0	–	65.0
TPU-IL65(MB)	35.0	–	65.0
TPU-CNT5.0(MB)	95.0	5.0	–
TPU-CNT0.5	99.5	0.5	–
TPU-CNT1.0	99.0	1.0	–
TPU-CNT1.5	98.5	1.5	–
TPU-CNT2.0	98.0	2.0	–
TPU-CNT2.5	97.5	2.5	–
TPU-CNT3.0	97.0	3.0	–
TPU-CNT1.5-IL5	93.5	1.5	5.0
TPU-CNT1.5-IL10	88.5	1.5	10.0
TPU-CNT1.5-IL15	83.5	1.5	15.0
TPU-CNT1.5-IL20	78.5	1.5	20.0
TPU-CNT1.5-IL25	73.5	1.5	25.0
TPU-CNT1.5-IL30	68.5	1.5	30.0
TPU-CNT1.5-IL35	63.5	1.5	35.0
TPU-CNT1.5-IL40	58.5	1.5	40.0

Table S3. Extrusion temperature for the melt-spinning of the drawn and nondrawn extrudates.

Drawn extrudates (1.5 m min^{-1})	Temperature (°C)	Nondrawn extrudates	Temperature (°C)
vTPU	215		
TPU-IL20	170		
TPU-IL35	157		
TPU-IL50	140		
TPU-IL65	170		
TPU-IL65(MB)	155		
TPU-CNT0.5 to 3.0	210		
TPU-CNT1.5-IL5	200		
TPU-CNT1.5-IL10	210		
TPU-CNT1.5-IL15	210		
TPU-CNT1.5-IL20	180	TPU-CNT1.5-IL20(ND)	180
TPU-CNT1.5-IL25	180	TPU-CNT1.5-IL25(ND)	180
		TPU-CNT1.5-IL30(ND)	170
		TPU-CNT1.5-IL35(ND)	170
		TPU-CNT1.5-IL40(ND)	170

Enlarged views from the differential scanning calorimetry (DSC)

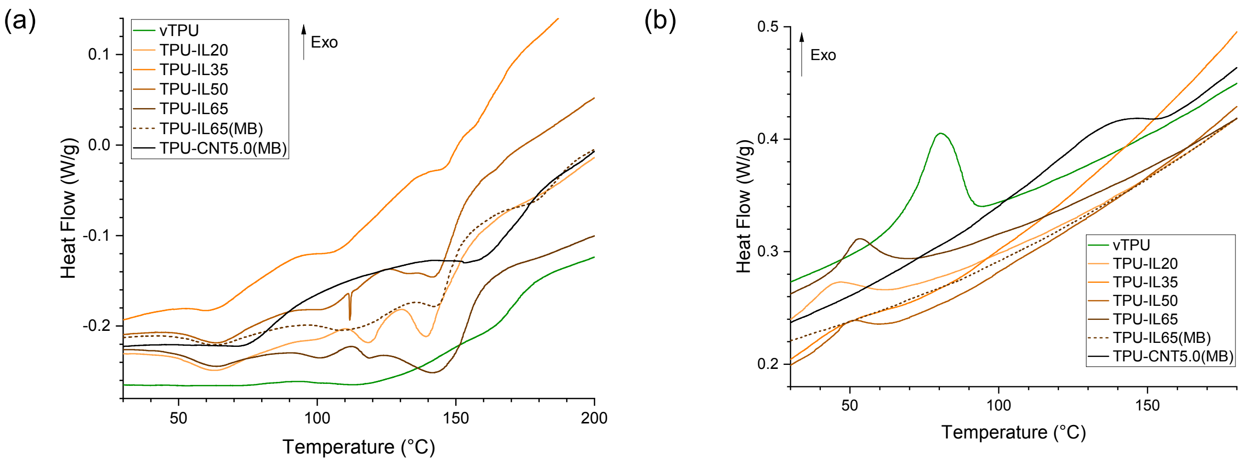

Figure S1. Enlarged views on the DSC curves of (a) the endothermic peaks during heating and (b) the crystallization peaks during cooling.

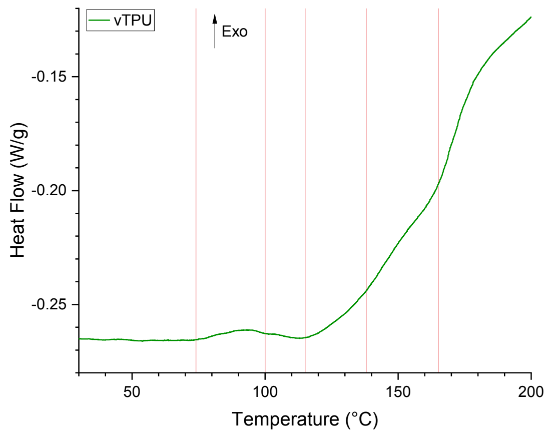

Figure S2. Enlarged views on the DSC curve of virgin TPU with indication of the endothermic events during heating.

Thermal analysis of the parallelly prepared TPU-IL65(MB) pellets

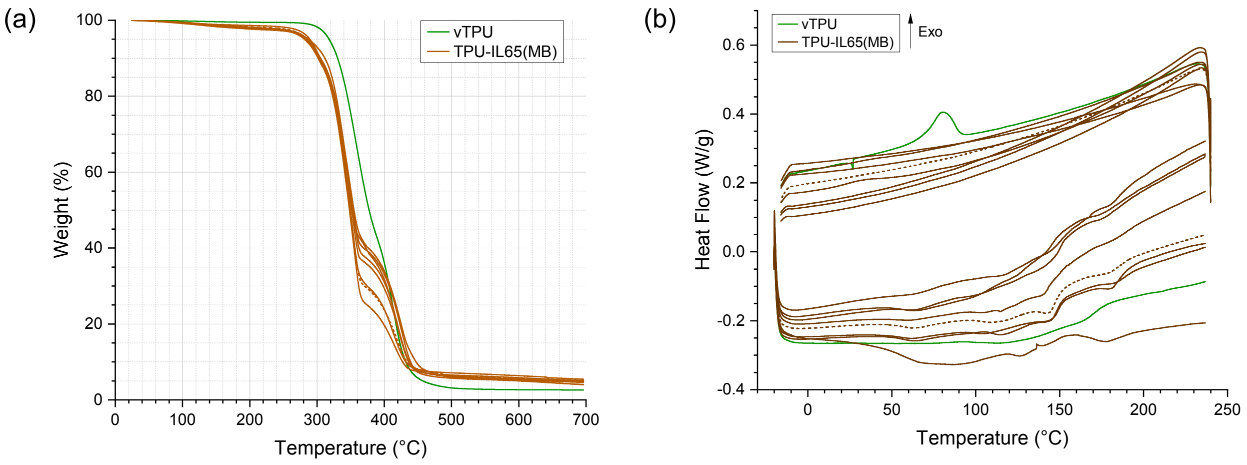

Figure S3. Results from (a) TGA and (b) DSC of eight parallelly prepared TPU-IL65(MB) pellets. For the peak analysis of the DSC curve, only the dashed curve was used for the peak analysis.

Scanning electron microscopy (SEM) images and energy dispersive X-ray (EDX) maps

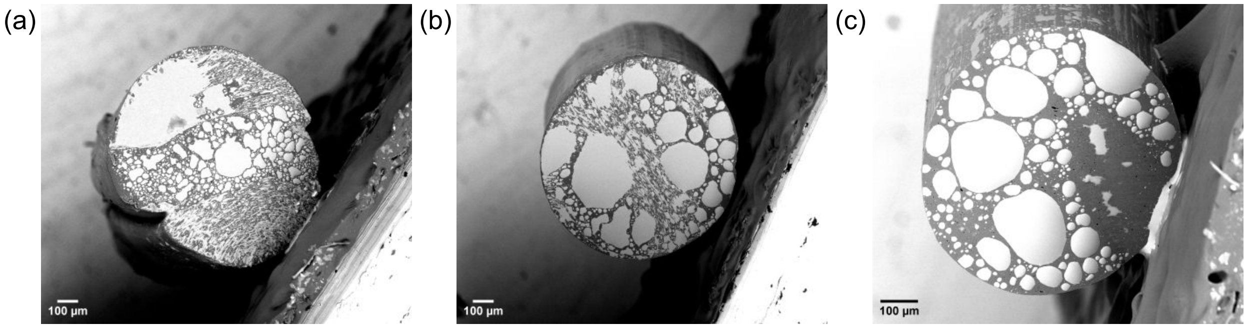

Figure S4. (a) Back-scattered electron images of the cross-sections of TPU-IL35, (b) TPU-IL65, and (c) TPU-IL65(MB).

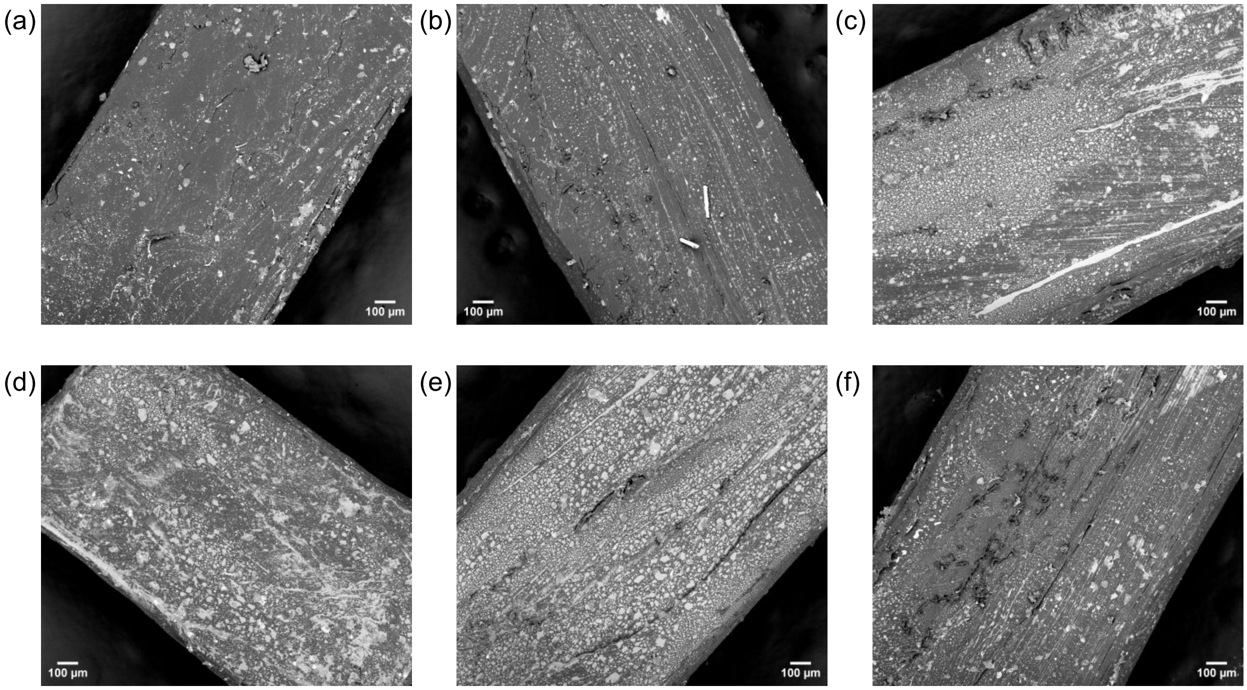

Figure S5. Back-scattered electron images of the longitudinal sections of the CNT-containing samples (a) TPU-CNT1.5, (b) TPU-CNT1.5-IL5, (c) TPU-CNT1.5-IL10, (d) TPU-CNT1.5-IL15, (e) TPU-CNT1.5-IL20, and (f) TPU-CNT1.5-IL25.

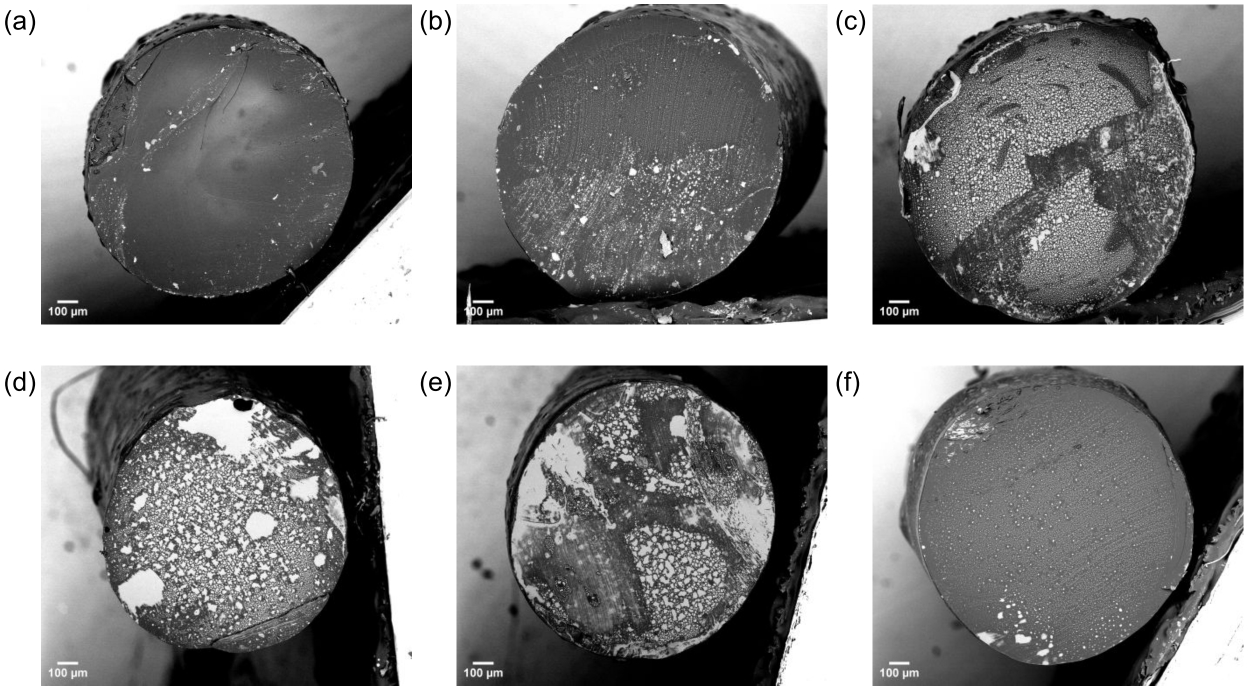

Figure S6. Back-scattered electron images of the cross-sections of the CNT-containing samples (a) TPU-CNT1.5, (b) TPU-CNT1.5-IL5, (c) TPU-CNT1.5-IL10, (d) TPU-CNT1.5-IL15, (e) TPU-CNT1.5-IL20, and (f) TPU-CNT1.5-IL25.

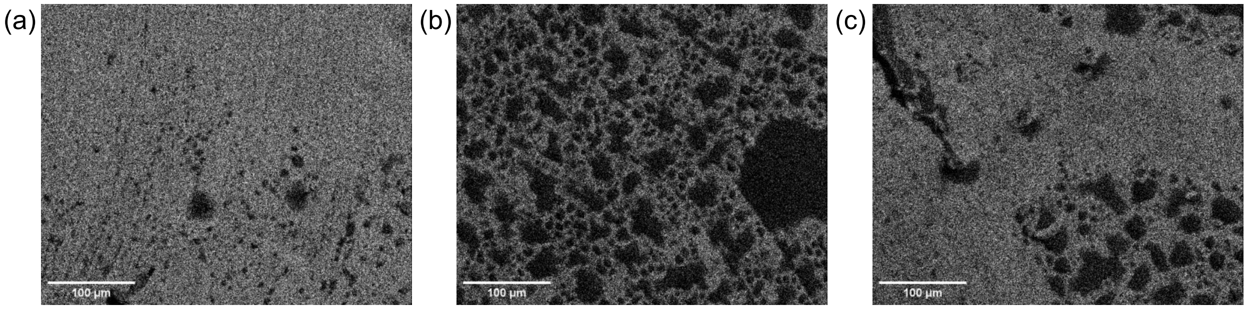

Figure S7. Close-up carbon EDX maps of the cross-sections of the CNT-containing samples (a) TPU-CNT1.5-IL5, (b) TPU-CNT1.5-IL15, and (c) TPU-CNT1.5-IL20. Brightness and contrast were increased to apply the following color pattern: black for pixel value 0, grey for 1, white from 2 to 8.

Close-up of the stress-strain curves

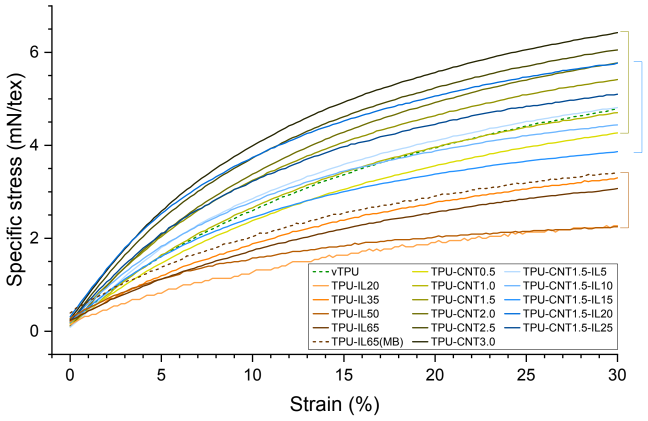

Figure S8. Close-up of the stress-strain curves on the first 30 % of strain.
1 mN tex^{-1} is equivalent to 1.12 MPa for virgin TPU.

Additional information and plots from the electrochemical impedance spectroscopy.

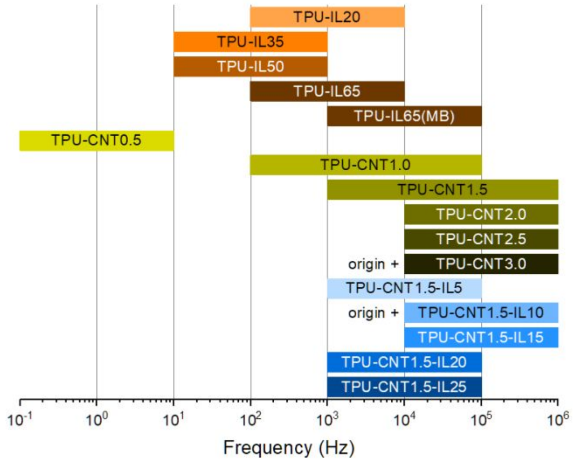

Figure S9. Range of frequencies selected for the semicircle fittings
in the Nyquist plots from the electrochemical impedance spectroscopy.
The origin was also added when it would otherwise result in an inaccurate fit.

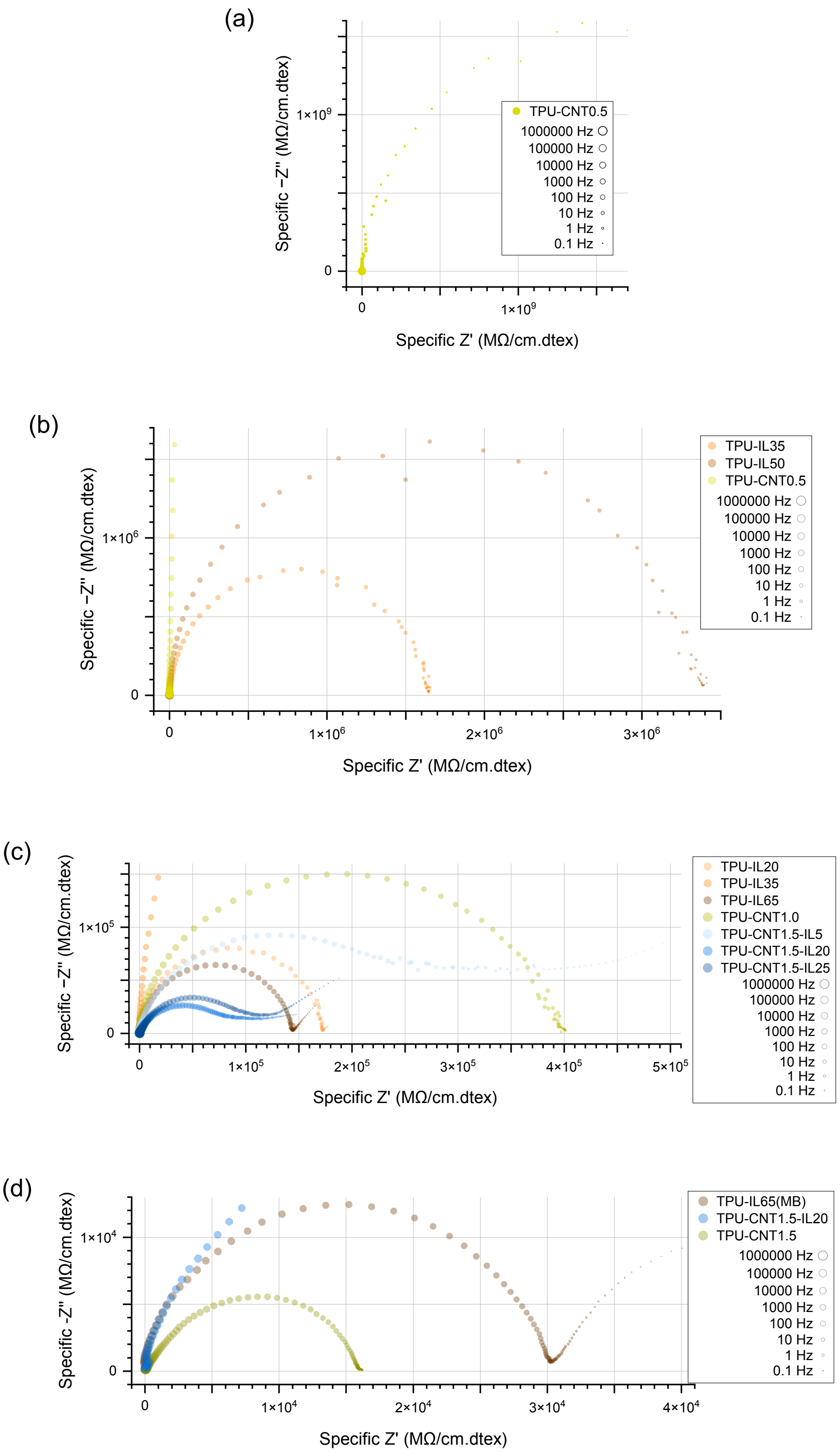

Figure S10. Specific Nyquist plots with different scales around (a) 10^9, (b) 10^6, (c) 10^5, and (d) 10^4 MΩ cm^{-1} dtex. (continued next page)

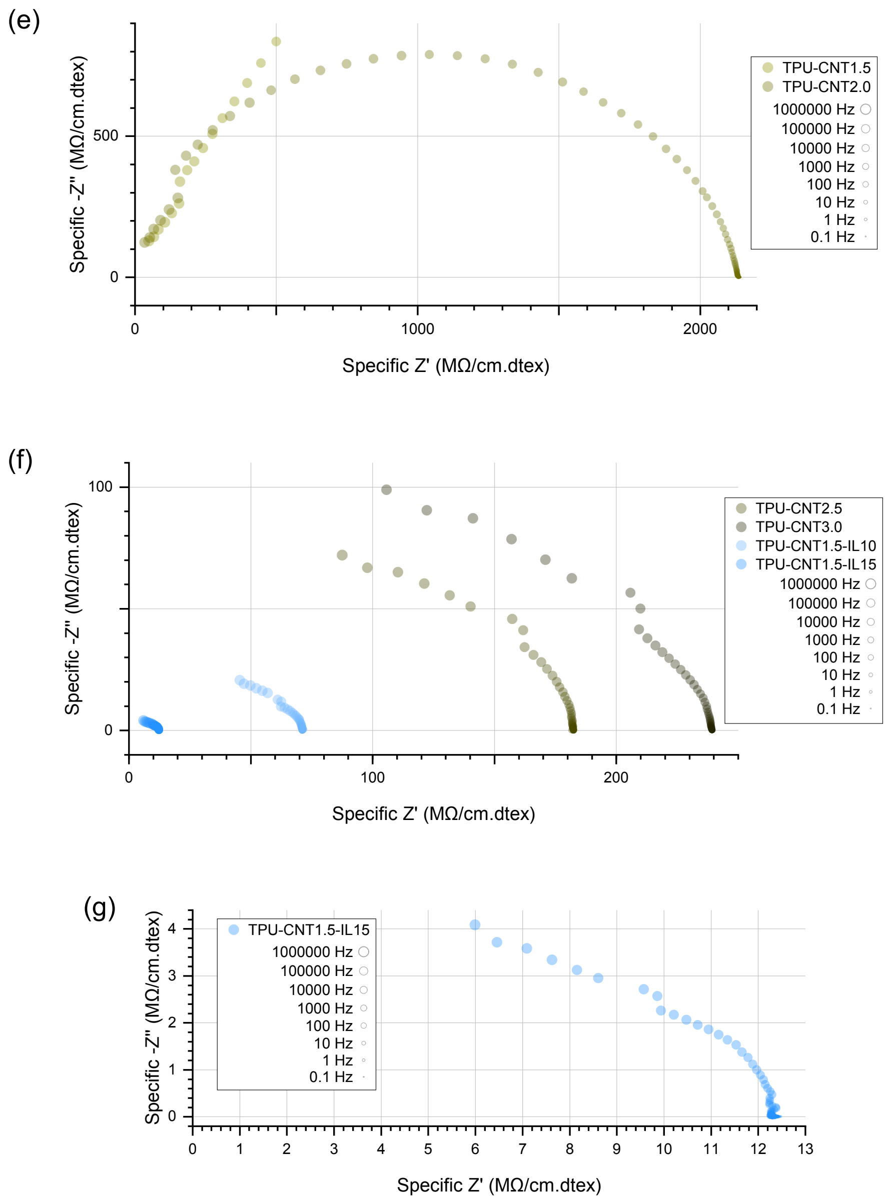

Figure S10. Specific Nyquist plots with different scales around (e) 10^3, (f) 10^2, and (g) 10 MΩ cm^{-1} dtex. (continued from previous page)

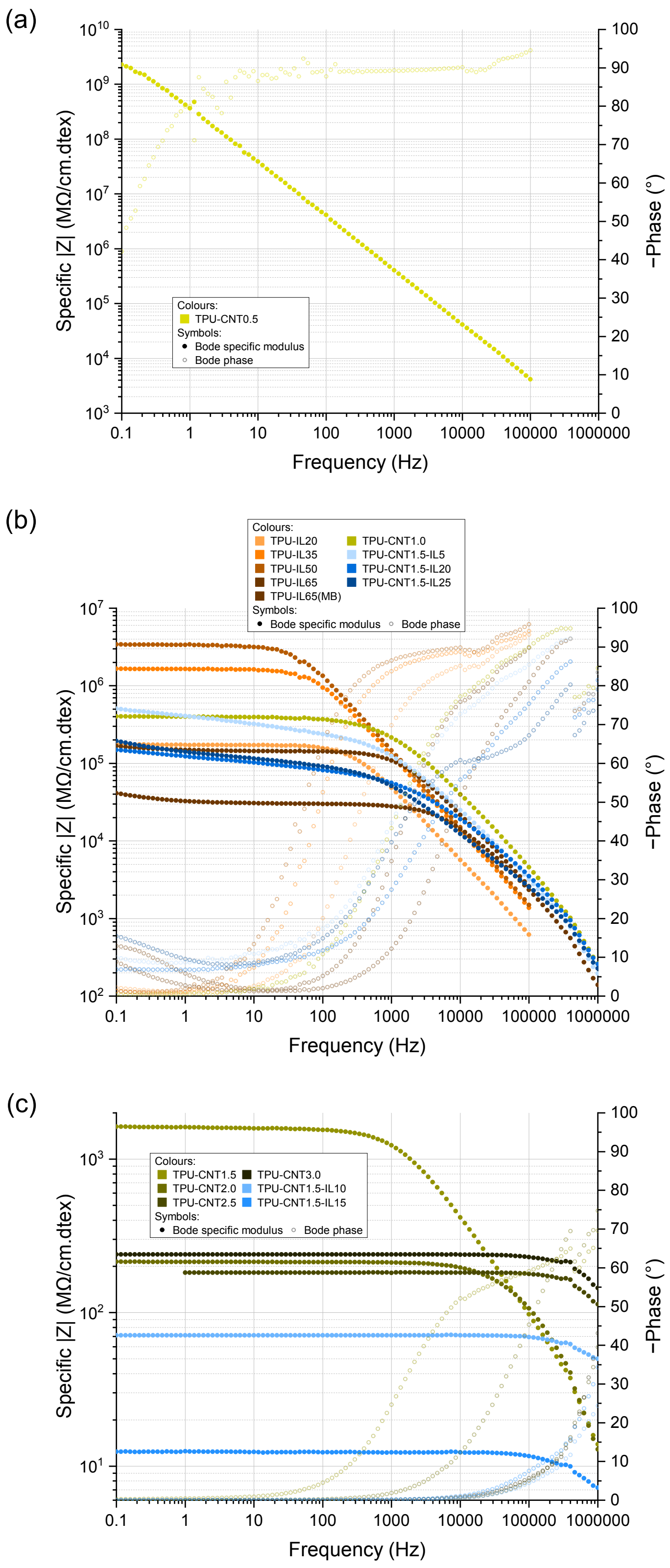

Figure S11. Specific Bode plots at different scales, with the magnitude of the specific impedance up to (a) 10^{10}, (b) 10^{7}, and (c) 2×10^{3} MΩ cm^{-1} dtex.

Cyclic voltammograms from the cyclic voltammetry (CV)

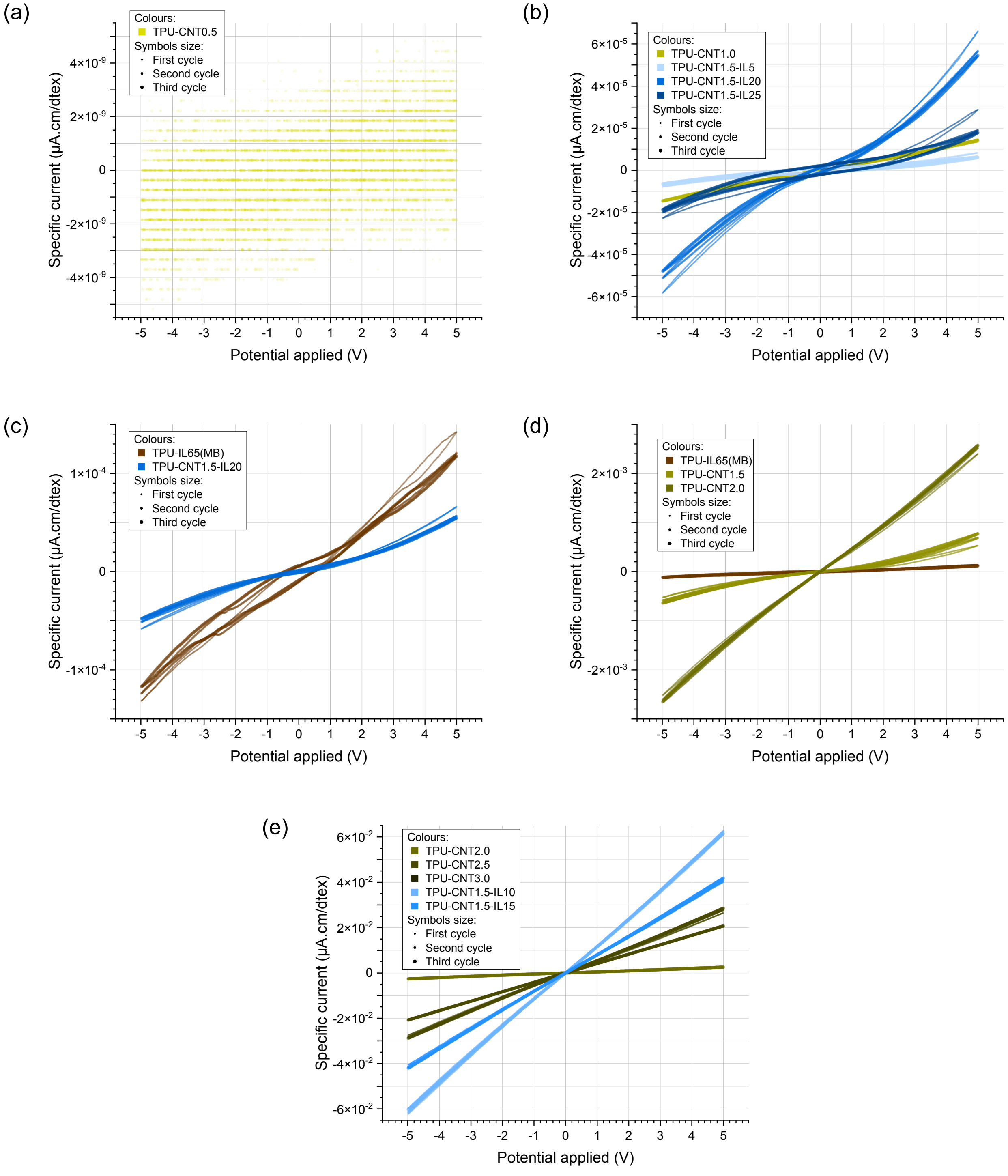

Figure S12. Cyclic voltammograms with different scales around (a) 10^{-9}, (b) 10^{-5}, (c) 10^{-4}, (d) 10^{-3}, and (e) 10^{-2} μA cm dtex^{-1}.
